# Reviewing the Structure–Function Paradigm in Polyglutamine Disorders: A Synergistic Perspective on Theoretical and Experimental Approaches

**DOI:** 10.3390/ijms25126789

**Published:** 2024-06-20

**Authors:** Nastasia Sanda Moldovean-Cioroianu

**Affiliations:** 1 Institute of Materials Science, Bioinspired Materials and Biosensor Technologies, Kiel University, Kaiserstraße 2, 24143 Kiel, Germany; nac@tf.uni-kiel.de; 2 Faculty of Physics, Babeș-Bolyai University, Kogălniceanu 1, RO-400084 Cluj-Napoca, Romania

**Keywords:** polyglutamine disorders, CAG expansion, neurodegeneration, aggregation, structure–function, protein dynamics, protein function

## Abstract

Polyglutamine (polyQ) disorders are a group of neurodegenerative diseases characterized by the excessive expansion of CAG (cytosine, adenine, guanine) repeats within host proteins. The quest to unravel the complex diseases mechanism has led researchers to adopt both theoretical and experimental methods, each offering unique insights into the underlying pathogenesis. This review emphasizes the significance of combining multiple approaches in the study of polyQ disorders, focusing on the structure–function correlations and the relevance of polyQ-related protein dynamics in neurodegeneration. By integrating computational/theoretical predictions with experimental observations, one can establish robust structure–function correlations, aiding in the identification of key molecular targets for therapeutic interventions. PolyQ proteins’ dynamics, influenced by their length and interactions with other molecular partners, play a pivotal role in the polyQ-related pathogenic cascade. Moreover, conformational dynamics of polyQ proteins can trigger aggregation, leading to toxic assembles that hinder proper cellular homeostasis. Understanding these intricacies offers new avenues for therapeutic strategies by fine-tuning polyQ kinetics, in order to prevent and control disease progression. Last but not least, this review highlights the importance of integrating multidisciplinary efforts to advancing research in this field, bringing us closer to the ultimate goal of finding effective treatments against polyQ disorders.

## 1. Introduction

To a great extent, diseases showcase a tight interplay between biomolecular architecture and pathological effects. Intricate configurations dictate both function (in healthy conditions) and dysfunction (in diseases). For instance, Alzheimer’s disease is characterized by excessive accumulations of amyloid-β plagues in the brain, triggering disruptive communications between neurons. However, in normal conditions, amyloid-β proteins have essential roles in neurons function and integrity. The function vs. dysfunction scenarios have one common nominator: in health, all molecular structures, in any form, work properly as long as cells are able to recycle and manage them. Once this physiological process is obstructed, the protein structures become harmful, by disrupting cellular function and inducing apoptosis. In the end, it is this loss of normal function that leads to the progression of various neurodegenerative diseases.

Whether in the realm of proteinopathies or within broader biological contexts, the shape and structure of molecules exert profound influence, since molecular architecture embodies the essence of intracellular complexity. The structure–function correlation, deeply embedded into molecular frameworks, governs the regulation of intracellular biochemical processes. Moreover, the dynamic behavior of molecular assemblies, including aggregation kinetics, adds another layer of complexity to this narrative. Hence, to understand these interdependencies’ involvement in diseases of all kinds, it is imperative first to recognize the relevance and impact of (bio)macromolecular shapes and structures, as well as their corresponding structure–function relationship, and their dynamic patterns. Ultimately, understanding these correlations unveils the essence of structural/molecular biology and biomolecular physics, while offering compelling opportunities for therapeutics discovery and unlocking the mysteries behind specific pathologies. 

Although the homorepeat sequences within full-length proteins are unsophisticated, or one could say ‘unusually simple’ segments of identical, reiterated, single amino acid residues, in the early 1990s, scientists identified their fundamental roles in biological activities (besides serving as linkers between multiple functional domains) including signaling pathways—underlying the proper mechanisms of various basic cellular processes such as cell division, differentiation, and/or development [1,2,3]. These homorepeat activities range from facilitating subcellular localizations and assembly to mediating protein interactions across various cellular pathways. 

The phenotypic diversity of homorepeat sequences is linked to tract length variations. Also, the polymorphic traits are observed among populations of the same organisms, and within different cell types, revealing the homorepeats’ instability and high propensities for cytotoxic behavior [4,5,6]. However, the exact origin of these genetically harmful and unstable tendencies remains unknown. Additionally, an increasing amount of evidence shows that abnormally extended tracts are considerably involved in human diseases, particularly in inherited neurodegenerative disorders. This involvement, solely based on functional versatility, is actually linked to the homorepeats ability to adopt heterogeneous configurations (structural assemblies that can adopt various shapes such as α-helices and β-sheets imposing a significant role in neurodegenerative diseases’ development and progression). Lastly, these structural configurations are known to be influenced by the local cellular environment (e.g., local pH and the presence of lipids), repeat composition and length, and undoubtedly by the nature of the flanking regions [3,7].

These homorepeats predominantly consist of amino acids like glutamic acid (polyE), proline (polyP), alanine (polyA), serine (polyS), leucine (polyL), glycine (polyG), glutamine (polyQ), histidine (polyH), aspartic acid (polyD), lysine (polyK), and threonine (polyT). Undoubtedly, their diverse roles are linked to the specific physicochemical properties of each amino acid. Generally, they modulate protein functionality, particularly in protein–protein interactions (PPI) that are crucial for gene regulation and signaling processes. Notably, proteins containing polyQ are disproportionately represented as central connectors in PPI networks [8]. It is also important to note that homorepeat sequences are predominantly hydrophilic; however, tracts of large hydrophobic residues were also identified within transmembrane regions of cell membrane proteins. The hydrophilic stretches clearly have lower tendencies to aggregate when compared to the hydrophobic ones. Interestingly, it has been demonstrated that it is not the homorepeat self-assembly which is causing toxicity in cells, but the property of homorepeat stretches to form highly stable aggregates of extended sizes [3]. 

Taken together, the structural complexity is built upon mixtures of structural subunits that govern the dynamic responses of biomolecular structures under specific environmental conditions. These kinetic responses are what finally dictate the biomolecules’ function. Hence, being able to understand and to characterize the structural changes occurring within homorepeat sequences such as polyglutamines (polyQs), and therefore to determine the polyQ’s dynamic properties, one should also be able to infer the functional traits of polyQs, and even of similar structures in different organisms. The well-known fact that protein structure has an immediate impact on the way the protein behaves stands beyond any doubt. However, understanding the homorepeat properties based on their sequence, structure, and dynamics may not be achievable via separating the homorepeat function, or its toxic trait, from the remaining part of the host proteins. Moreover, one should take into account that the interaction network’s complexity affects not only the function of individual proteins, but also their structural behavior. So once again, the structural relevance of a particular protein becomes trapped within ample coevolution networks formed between a myriad of interacting partners. 

The herein review paper discusses the nuanced interdependence between molecular architectures and pathological consequences of polyQ disorders, with the aim to cover the alterations of polyQ tracts and their host proteins, in terms of structure–dynamics–function, responsible for the formation of characteristic aggregates and neuronal inclusions. Additionally, this review offers an unconventional perspective by emphasizing the significant value of synergistic approaches existing in the field. Lastly, this review aims not to exhaustively cover every published paper, but rather to capture the central motifs, key concepts, unanswered questions, and ongoing efforts towards finding effective treatments against polyQ disorders, and even neurodegeneration within expanded reach. 

## 2. Fundamental Traits in the Study of PolyQ Diseases 

### 2.1. Shape and Structure

The primary focus in structural biology revolves around a thorough understanding of the structure–function relationship of macromolecules. To fully understand how proteins work, it is essential to gain insights into their structural hierarchy. The primary protein structure corresponds to the actual amino acid sequence. Due to amino acids inter- and intramolecular forces, the secondary structural components are formed (e.g., α/β-helices, α/β-sheets, bends, turns, random coils). Subsequently, the three-dimensional (3D) shape of proteins is dictated by their tertiary order of structure. At this stage, multiple protein strands interact with each other via ionic and hydrogen bonds (H-bonds), disulfide bridges, and hydrophobic/hydrophilic contacts. And finally, the quaternary structure represents the protein’s active form in a highly packed shape that associates several protein chains/subunits, with specific primary, secondary and tertiary components. These subunits form functional oligomers that are held together mainly through non-covalent intermolecular forces (H-bonds and van der Waals interactions) or covalent interchain disulfides [9]. The oligomeric states can easily undergo drastic and rapid conformational changes that might ultimately affect distinctive biological activities. 

Proteins’ structures have been determined using multiple experimental methods, including X-ray crystallography, Cryo-EM [10], and NMR [11,12]. Nowadays, cryo-EM has clearly emerged as a leading technique for elucidating macromolecular structures at near-atomic resolution, while also providing valuable insights into the energetic and conformational landscapes of macromolecules, thus playing a crucial role in addressing the challenge of conformational heterogeneity in protein structure–function resolution. However, X-ray crystallography excels at providing precise atomic coordinates of structures with sizes under a few hundred kDa, also being better equipped to bear high-resolution dynamic insights as a function of time, temperature, and pressure. Hence, it remains unclear which of the two techniques should be portraying the future of structural biology [13]. Just as important, NMR experiments determine which atoms are in the vicinity of a given atomic nucleus. With this method, structure determination becomes highly challenging for proteins larger than 50 kDa since they tumble (e.g., rotate, vibrate, translate) too slowly, involving rapid nuclear relaxations and low detection sensitivities, leading to substantial constraints in protein structure determination. A combined approach using NMR and EM data, as introduced by Gauto et al., overcomes the limitations of each method [14]. 

In 1963, Ramachandran et al. pioneered the 2D investigation of the torsion angles of amino acids, also known as φ-ψ mapping in a protein sequence, describing the proteins’ backbone conformations. This metric of experimental structural models quantifies the residues belonging to the ‘outlier’, ‘allowed’, and ‘favored’ regions [11,15]. Ramachandran plots can be used to theoretically assess which conformations, or values of the ψ and φ angles, are possible within the protein residues and to indicate the empirical distribution of multiple data points observed in a single structure; particularly useful for macromolecular structure validation in 3D models [15].

But what causes changes in the structure of a protein? Aside from the obvious interplay between intra- and intermolecular forces within and between amino acids, proteins can also alter their shape via specific interactions with other interacting partners, following transitions from inactive states into active ones. Apart from the arrangements of the amino acid residues, intermediate phases caused by hydrophobicity-to-charge ratios can lead to structural modifications. Regardless, these structural changes are key enablers of proteins to carry out their designated functions within cells. Changes in temperature, pH, and/or exposure to chemicals can lead to significant alterations in protein assemblies. In these cases, the proteins might turn dysfunctional without even encountering changes in their amino acid sequences. 

Conversely, a mutation in the amino acid sequence can alter protein stability and folding patterns, affecting its interactions with other structures, and therefore its overall function. Mutations might occur far from functional sites but still impact protein activity. In the same context, even though the mutant proteins might structurally resemble the wild-type (wt) ones, the mutants’ topologies could present alterations at sites distant from the specific sites of perturbation/mutation. This aspect has been extensively discussed in recent years under allosteric signaling [16,17,18,19,20,21,22]. Hence, upon any disease-causing perturbation in a system, such as genetic mutations, variations within the protein structural network (PSN) are known to initiate ‘allosteric changes in functional sites and elsewhere’ [22]. Alternative models for resolving structural and dynamical perturbations within the protein context are mentioned and briefly outlined in the forthcoming subsection.

### 2.2. Structural Networks and the Structure–Function Paradigm

The function of a protein depends on its structural flexibility based on the unique (re)arrangements of the atomic interactions. Under specific environmental conditions, the protein’s intrinsic flexibility is reflected by the empirical changes in its secondary and tertiary structure, which are dominated by the protein’s dynamics and may be classified as either subtle changes in the side chains or larger variations within the protein’s backbone configuration. The global topology of proteins can be studied using the previously mentioned Ramachandran plot, or through understanding of the neighboring side chains interactions Moreover, the inter-residue interaction networks, which are widely used for homology detection and protein structure predictions, are crucial for comprehending the proteins’ functional behavior [11,23,24,25,26,27].

Due to many interconnected dependencies in protein topologies, scientists represented the intra-protein interactions as interaction networks, where the spatially proximal residues are represented as nodes, and the edges represent the connections between those residues. This node-edge model is currently known as protein structural network (PSN) and is extensively employed in the protein structural analysis centered on geometries, topological distances, charges, solvent accessibility, and energies. PSNs are not only applicable for intra-protein models, but also for significantly larger interacting systems [28,29,30,31,32].

Since the structural similarities between proteins may also represent their functional similarities, in 2020, Newaz et al. proposed the first network-based protein structural classification (PSC) framework. The authors concluded that by integrating distinctive protein features, the PSC’s accuracy clearly improves, particularly for ordered graphlets, due to the integration of both 3D structural and sequential information [33]. An alternative approach for protein network representation is the protein contact network (PCN) which is based on spatial distances or interaction energies between amino acids (defined as nodes), allowing for a detailed investigation of proteins from several structural-functional perspectives [34,35,36,37]. These perspectives rely on pivotal residues identification involved in, e.g., protein stability and/or dynamics, folding kinetics, enzymatic activities, allosteric regulations, and signal transduction [29,38,39,40,41,42,43]. Different types of network-based analysis are known to facilitate the quantitative/qualitative examination of residue–residue interactions in single-chain models and protein complexes. The constructed networks (e.g., atom pair contact, centroid, and interaction-strength type of network) are notably useful in folding pattern analysis of structural repeats, protein domain identifications, side chain clustering, binding cavities recognition, and analysis of allosteric communication, physicochemical properties and protein thermo-stability [44]. Therefore, the PSN yields valuable insights into protein structure and stability by establishing the amino acid side chains involvements in retaining the proteins’ unique topologies. 

Indeed, proteins and PPIs stability is a prerequisite of function, and therefore a target for natural selection which is the key mechanism of evolution. Moreover, stability correlates with increased structural fitness and robustness and enhances the proteins’ capacity to undergo functional changes [45]. Conversely, these functional changes might actually require certain structural alterations. For example, the catalytic residues of an enzyme or the binding hot spots (a.k.a ‘functional epitopes’) on the protein surfaces, which are sites not associated with the protein scaffold’s formation, are also essential for protein function. Other contrasting examples include the inherently disordered structures whose functions emerge from many different conformations, such as structured globules, collapsed and extended ensembles, and notably, from their drastic transitions between these conformations. Also, studies have shown significant structural differences between natively unfolded proteins (with high propensities for helical conformations) and the denatured unfolded proteins (with mixed amounts of β-structures and α-helical configurations). All these structures are known to carry out essential biological functions, with pivotal roles in signaling and regulatory pathways throughout protein–protein, protein–ligand, and protein–nucleic acid type of interactions [46]. Regarding the protein sequence content, the major influence of the amino acid sequences in the structural and, consequently, the functional context of proteins is one of the main aspects of interest in the following sections. 

In 2018, Fuxreiter discussed another theoretical perspective based on stochastic models used to obtain the functional basis, sequence motifs, dynamical features (from local motions to large-amplitude collective ones), and conformational heterogeneity of biomolecules [47]. In a ‘fuzzy inference system’, the input parameters describe the sequence motifs or conformational space (macrostates and microstates), whereas the output elements define distinctive biological activities of the system. After the fuzzification of the input, knowledge-based logical rules (if-then), which can be derived from neuronal network algorithms (NNA), were applied. The output’s defuzzification ultimately correlated with the most likely activity underlying certain conditions, while accounting for other ‘promiscuous activities’. The challenges in defining the functions within the *fuzzy* formalism are rigorously addressed by M. Fuxreiter [47].

But what are the concrete ambiguities of the classical sequence–structure–function paradigm? First, it assumes that a given sequence settles a well-defined assembly owning a specific function. Second, it can not account for the multiple (and simultaneous) protein activities. Third, the heterogeneous assemblies are based on mixtures of configurations with distinctive functional purposes (e.g., in signaling activities). And fourth, some protein structures might present weak sequence dependencies [48,49,50]. On these grounds, the same ensemble can, therefore, undertake several functions—known as functional promiscuity. Furthermore, the same sequence may contain multiple functional conformations (functional domains), which may initiate multiple interactions—known as conformation and interaction heterogeneity. And lastly, a large variety of sequences may actually encode the same conformational ensemble—known as sequence redundancy [47]. Hence, the protein dynamic adaptation is facilitated by all of the previously mentioned ambiguities and redundancies [47,48,49,50,51]. 

Discoveries from the last decade also show that functional encoding does not correlate with the sequence itself, alternatively any changes in the sequence would trigger changes in function. This comes in agreement with other similar perspectives, in which the functional basis can not be assigned via a unique structure/assemble [52,53,54], but rather via sets of multiple structures. Therefore, the protein function might be resolved only by sets of atomic motions describing many structural transitions.

### 2.3. When Dynamics Comes into Play

In the simplest terms, dynamics comes into play when the structural patterns are no longer ‘able to cover’ for the functional ones, or to be more precise: when space and time matter. At this point, the sequence–structure–function paradigm becomes somewhat limited. It is generally considered that once the amino acids within protein sequences are tightly linked and threaded together, proteins reach their final shapes via intramolecular bonds, which prompt the proteins’ folding behavior. This scenario is genuine, however, in theory and merely for isolated structures. In a real environment, proteins hardly work alone. As known, the cytoplasmic matrix is considerably crowded and occupied by many other macromolecular structures. Hence, these structures can easily interact with, e.g., partially or wrongly folded proteins, triggering a myriad of inappropriate alliances and reactions. The faulty reactions eventually interfere with the proper folding mechanisms and give rise to the formation of large intracellular aggregates. Ultimately, these insoluble aggregates cause cellular toxicity.

Protein dynamics incorporates atomic motions pinpointing different mechanisms of action and occurring within diverse timescales and amplitudes. These motions largely depend on the type of systems involved and the environmental conditions. The principal motions in protein dynamics include bond vibrations (fs-ps timescale), side chain rotations (ps-ns), backbone fluctuations (ns), loop motions (ns-ms), ligand dissociation and/or association (ns-µs), slow movements of collective (domain) translocations (>µs), catalytic events (µs-ms), followed by the most ample structural modifications during protein folding/unfolding processes and allosteric changes (hrs). An efficient and common approach of identifying and quantitatively exploring the coordinated atomic motions within protein networks is by using the principal component analysis (PCA) method [55]. In terms of atomic interactions, residue networks can identify different non-bonded contacts such as H-bonds, van der Waals (vdW), salt bridges, π–π and cation–π interactions, Coulombic, as well as arginine-arginine interactions (due to its charged groups that may interact with both positively and negatively charged side chains of the amino acids). Moreover, for the assemble-based approaches conformational transitions can also shed light on the protein dynamics, highlighting the relevance of protein side chain and backbone flexibility via Cα atoms measurements [55]. These techniques are usually attended by local energy landscapes for quantifying the association between structural, dynamical, and functional traits of proteins. 

To account for the interacting biological systems’ complexity, experimental studies combined with molecular simulations and network-based models are widely considered as well-suited procedures to decipher the couplings between protein dynamics and function [56,57,58,59,60,61,62,63]. For instance, the global mode analysis, dynamics network models (DNM), elastic network models (ENM), and protein energy networks (PEN) serve the purpose of tracking multiple scale dynamics in large biomolecules with further identifications of the residual involvements in biological activities. Other integrative methods, such as perturbation response scanning (PRS) and network evolution models, aim to integrate the impact of gene variants on biomolecules features that might impede proteins from working properly. Alternative approaches are able to associate scale of motions with different types of activities [64]. Also, knowing the amino acid properties that have a direct impact on functional tolerance within particular conditions, represents a key factor in elucidating the hallmarks of functional dynamics in health and disease [52]. In this view, many proteins might share the same structural features, however involving significantly different motions in order to execute their functions. Accordingly, protein dynamics is generally assumed to be ‘neighboring related’. 

Indeed, the neighborhoods of each constituent amino acid of proteins, on average, trigger a moderate number of intramolecular interactions. Inside all proteins, each of the adjacent amino acid is surrounded by different numbers and/or types of neighbors. The neighbors are then able to describe the amino acid’s spatial distribution of the protein structure, and to account for the occupied and unoccupied spaces between amino acids, ‘where atomic motions can take place’ [21,52,65]. In computational biology, the amino acid network (AAN) is an established method to describe the amino acid neighborhoods within a protein, in which the nodes are defined as amino acids, whereas the links are defined as the actual interactions (based on atomic proximity) between the residues. As a particularity, in order to investigate the space occupancy at different spatial scales (for tracking functional dynamics), the amino acid neighborhoods are computed at varied cutoff distances [65,66,67]. 

In order to properly acknowledge protein dynamics based on structural contributions, various experimental techniques such as NMR, cryo-EM, small-angle X-ray scattering (SAXS), mass spectrometry (MS), atomic force microscopy (AFM), and single-molecule fluorescence resonance energy transfer (smFRET) are widely used to study pH-, temperature-, mutation-, protein- or ligand-induced conformational modulations [68]. Moreover, in recent decades, significant advances in machine learning (ML) methods and artificial intelligence (AI) have also been made in order to depict essential information from large protein structural datasets and to improve the sampling of drastic conformational events in protein folding/unfolding and dynamics. The scope of algorithms is to extract complex structural patterns and hidden structure–dynamics relationships. Just as important, the physics-based methods are suitable for designing energy functions that describe protein dynamics from one conformational state to another (e.g., from an unfolded to a folded state). Approaches from the first-principle atomic force fields (FFs) to simplified coarse-grained (CG) representations are widely used nowadays for encoding the physical principles related to, e.g., energy landscape theory in protein folding. Hence, neural networks are able to integrate these energy functions for multibody interacting systems analyses [69,70,71]. 

In terms of folding kinetics, it is well known that folding rates, mechanisms, and functional motions rely on protein native topologies. However, the conflicting protein interactions—also known as *frustrations*—are what dictates how fast biomolecules can explore their conformational landscapes. As stated by Kluber et al. [72], ‘frustration is a central concept’ in protein folding that may be correlated with ‘slow reconfiguration dynamics’. Additionally, the authors explored the non-native interaction effects on reconfigurational and folding dynamics for different protein sizes and topologies. For instance, their study show that—depending on the strength of non-native interactions—α-helical proteins have rather compact misfolded ensembles, and therefore slower reconfiguration dynamics than the β-sheet structures of comparable sizes. Accordingly, these findings provide additional insights into the non-native heterogeneity, the role of frustration in protein misfolding and ‘why some proteins appear more frustrated than others’ [72].

## 3. PolyQ Expansions and Neurodegeneration

PolyQ regions are the most frequent homorepeat sequences characterized by consecutive stretches of glutamine (Q) amino acids, found in eukaryotic proteins [7,73,74], and were first characterized in 1989 at the N-terminal domain’s level of the human androgen receptors (ARs) [75]. Three years later, the polyQ expansions were, for the first time, associated with X-linked spinal and bulbar muscular atrophy (SBMA) disease [76,77]. 

There are approximately 4808 human proteins consisting of polyQ regions; however, only a few of them are associated with pathological phenotypes [78,79,80,81,82,83]. The neurodegenerative diseases associated with polyQ expansions (elaborated in Table 1) include SBMA, spinocerebellar ataxia (SCA) type 1, 2, 3, 6, 7, 8 and 17, Huntington’s disease (HD), Huntington’s disease-like 2 (HDL2) and dentatorubral pallidoluysian atrophy (DRPLA). These types of disorders are described by abnormal expansions of the trinucleotide CAG [8,78,79,80,81,82,83] that are ultimately translated into enlarged, highly packed and hardly soluble polyQ stretches. It is worth noting that CAG repeat expansions can occur in either protein-coding exons or in untranslated regions [84]. Their pathological hallmarks are the accumulations of cerebral inclusion bodies, with the progressive apoptotic effects on the neuronal cells within disease-specific brain regions or spinal cords, leading to neurodegeneration and neurological impairment [85,86,87,88]. Depending on the disease, the pathological conditions outline an augmentation of CAG repeats above the mutational threshold of 21–40 Qs [80,88].

According to Totzeck et al. [89], who focused on investigating the impact of polyQs on protein structure, a structurally functional polyQ region requires at least four Q residues. Moreover, they observed that polyQ sequences were oriented towards the exposed side of the investigated (folded) protein structures, which further supports the highly debated function of polyQ in PPIs [79,89]. However, the polyQ-rich structures may also contain other inserted impurities, such as proline, histidine and leucine residues [90,91], due to the fact that these amino acids are coded by only one mutational codon apart from CAG (and CAA). Proline can be translated by similar codons including CCG and CCA, histidine is translated by CAT and CAC trinucleotides, whereas leucine can be coded by CTA and CTG. Hence, it has been shown that leucine and proline residues may significantly influence the polyQ aggregation processes, considering two divergent perspectives: leucine residues may promote the α-helical components’ evolution, while the proline residues (situated C-terminally) may reduce the polyQ’s tendencies to aggregate [92,93,94]. 

Regarding the intracellular effects of polyQ stretches, the high concentrations of compact shaped proteins are mainly involved in accelerated aggregations and cleavage processes, transcription dysregulations, mitochondrial dysfunctions and autophagy impairment [88]. To fully address these effects, the polyQ fragments are formed via hydrolysis reactions that breakdown the proteins into smaller polypeptides or amino acids—a process known as proteolytic cleavage [95,96,97]. The cleavage takes place through multiple proteases that speed up the formation of fragments with an increased propensity to form highly compact and toxic aggregates. Although the knocked spot inclusions are commonly rich in hardly soluble or even insoluble polyQ phases responsible for cerebral damages, the exact role of the proteolytic cleavage in polyQ diseases remains elusive [88]. In this context, further studies on the cleavage sites are mandatory for identifying the exact involved enzymes responsible for polyQ fragmentations, and whether any inhibition at this level might provide valuable insights for the therapeutic development against polyQ diseases. 

Protein quality control in cells is represented by specific autophagic processes (also known as macroautophagy), consisting of regulated mechanisms of cellular components removal and recycling [98,99]. For optimal central nervous system (CNS) functioning—in neuronal compartments—these processes break the misfolded proteins and/or other dysfunctional organelles. Therefore, any autophagy defects will undoubtedly influence the progression of various neurodegenerative disorders, with a dominant effect on the molecular mechanism of polyQ-related diseases [100,101]. As already known, the dynamic autophagy mechanism takes place through lysosomal degradation. In this catabolic pathway, double/multilayered membrane vesicles (autophagosomes) are being formed in the cytosol, enclosing macromolecules and organelles, therefore allowing for their degradation (by lysosomal hydrolases) after the vesicles’ fusion with lysosomes (autolysosomes) [102]. In pathological conditions, the post-mitotic neurons are highly dependent, therefore susceptible, on the basal autophagy mainly because misfolded/dysfunctional proteins/organelles are not able to mitigate, due to the fact that they can not replicate [103]. Moreover, the presence of cytosolic polyQ aggregates might suggest abnormalities in the cargo recruitment, therefore in the formation of autophagosomes, leading to failed processes of protein degradation [98]. However, is this autophagic impairment an ultimate result (as an effect) or a primary target for this type of disease? Within this context, the goal in this review paper is to offer a clearer perspective on autophagy-mediated degradation by emphasizing the autophagic, and consequently lysosomal perturbations involved in polyQ diseases. 

In regard to the bioenergetic ATP-dependent mechanisms, any deficit at the intracellular level would imply high-energy demands induced by cellular respiration [104]. Moreover, elevated oxidative stress is commonly found in polyQ diseases, as well as depletion of the intracellular ATP pool together with electron transport deterioration [101,105,106]. Hence, mitochondrial pathway dysfunctions are generally considered as a fingerprint for polyQ disorders, although the exact impact of these perturbed processes remains unclear. 

Interestingly, while the expression of most polyQ disease proteins is widespread, only particular brain regions are being affected. The selective vulnerability involves targeted cellular pathways with their corresponding target proteins, imposing high interacting affinities for polyQ structures in specific types of neurons [81]. 

But what brain-region-specific factors are actually accountable for determining this selective vulnerability? In 2014, Walker S. Jackson highlighted the complexity underlying the preferential damage distribution of the affected neurons. Starting from the well-known assertion that all neurodegenerative disorders are caused by structurally abnormal (misfolded) proteins, that eventually clusterize into aggregates [107], other proteins such as molecular chaperons, ubiquitin-binding structures, proteasomes or transcriptional coregulators, may interact with these aggregates [108,109,110], triggering high intracellular metabolic rates required for the damaged cellular components degradation and their repairs. The increased metabolic demand, as the brain ages, delineates a waterfall reaction responsible for further enlargements of the expanded polyQ tracts [81,107]. An alternative hypothesis is based on the actual location of the targeted neurons in the brain, suggesting that the neurochemical firing and the intrinsic cellular properties are not the only key parameters able to influence the neuronal vulnerability. Moreover, non-neuronal cells and metal ions [111,112,113] may also be involved in disease progression and may influence selective vulnerability, by triggering both positive and negative intracellular effects. However, due to many unsolved puzzles in this regard, additional research studies must be considered [107]. 

The involvement of both gain- (GOF) and loss-of-function (LOF) effects [114,115] represents an important feature of the neurodegenerative patterns related to polyQ diseases, and makes the development of effective targeted therapies even harder, particularly without an adequate understanding of the involved cytotoxicity mechanisms. 

Walker S. Jackson points out in his review article [107], perhaps the most relevant and intriguing aspects related to the role of aggregates in neuronal degeneration: (i) If some aggregates are benign, and others might actually be helpful for a proper neuronal development, where does the toxicity within polyQ aggregates come from? (ii) Is this toxicity a repercussion of the preformed clusters and should we consider it as a defense mechanism? And (iii) to what extent do identical aggregates cause contrasting damages, considering the previously discussed selective neuronal vulnerability? 

So far, scientists have agreed that the most common molecular feature of polyQ diseases is undoubtedly described by the sole existence of extended polyQ stretches at various locations within the genome [116]. More specific, the formation of abnormal secondary structural components, caused by slipped strand mispairing (SSM) processes, trigger (apart from the toxic aggregation itself) isolated intracellular events including mitochondrial dysfunction, oxidative stress, proteasomal and autophagy impairment, neuroinflammation, and potential toxic RNAs [85,117,118,119,120]. 

But how exactly do these formed aggregates ultimately impact the neuronal development? Although the exact cause of polyQ-protein aggregates inducing neurodegeneration still remains unknown [85,117,120,121,122], it has been demonstrated that all polyQ diseases share similar mechanisms of pathogenesis, and that CAG repeat expansions with their correlated conformational changes provide specific deleterious effects [115]. However, these protein-related diseases do not share similar amino acid sequences, therefore exhibiting distinctive secondary and ternary structural components, and consequently different dynamic behaviors and biological functions. A frequent explanation on this matter, is that ubiquitinated polyQ aggregates bind many other proteins such as transcription factors (TFs), proteasomes and chaperons, increasing the aggregation propensities through additional sequestration of other non-expanded proteins [115,117]. Also, the sequence context of the involved mutant proteins (including the flanking regions) might also influence the disease-specific neurodegenerative effects, therefore the protein aggregation itself is unlikely to be “an epiphenomenon event” [117]. Another plausible scenario is that the partial loss of the physiological protein functions might significantly affect a proper neuronal development and encourage the neurodegenerative processes [115,123]. To conclude this paragraph, the most probable event that accurately describes the polyQ diseases pathogenesis combines both causation—the polyQ expansion itself—and the consequences of the conformational changes associated with these expansions.

In terms of neurotoxicity, one hypothesis is that polyQ inclusions are key mediators for neuronal dysfunction, whereas alternative assumptions suggest that these inclusions are actually non-pathogenic and might also present protective roles via sequestering smaller, toxic polyQ structures. These contrasting hypotheses, supported by solid evidence, point out that polyQ tracts might be indeed protective in early stages of diseases, but harmful in later phases [81,124]. 

As previously mentioned in this section, proteins responsible for polyQ-related disorders have distinctive structural features, while providing different intracellular localization and functions. Considering that these disorders clearly share common clinical and neuropathological effects (see Table 1), the symptoms emerge at middle ages and worsen over 15–20 years, until death. As another common trait, an earlier disease onset is directly correlated with extended polyQ tracts, ultimately triggering the inclusion bodies’ formation in the brain. Finally, when polyQ disorders are passed on to the next generation, the symptoms may become more severe, at an earlier age and within longer polyQ tracts. This phenomenon is referred to as genetic anticipation [116]. 

**Figure 1:**
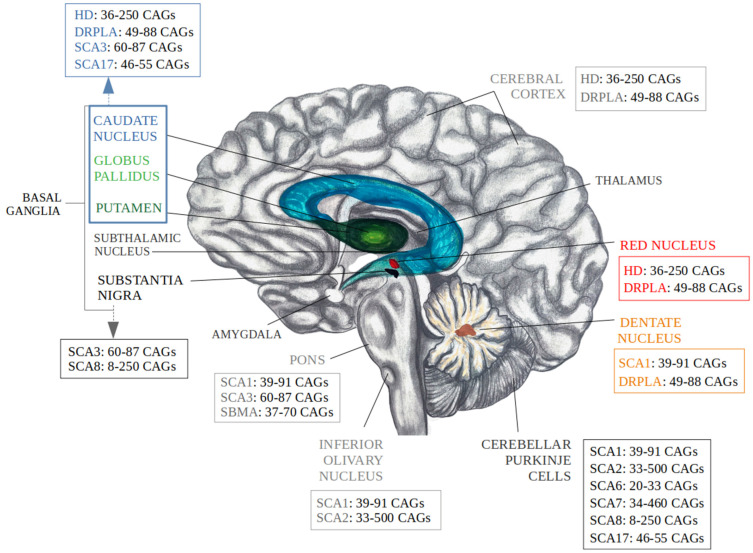
The affected brain regions in polyQ disorders and the corresponding pathogenic CAG repeat ranges. Personal sketch adapted from Naphade et al. [126]. The basal ganglia region is affected by HD, DRPLA, SCA3, SCA8 and SCA17, with CAG ranging between 8 and 250 repeats. Except from SCA3, all SCAs have projection areas within the cerebellar Purkinje cells with CAG ranges of 8–500 repeats. SCA1 also affects regions like dentate nucleus, pons, and inferior olivary nucleus with CAG repeats ranging between 39 and 91. HD also affects the cerebral cortex and red nucleus, while SBMA underscores an impact within the pons region with CAG range of 37–70 repeats. SCA—spinocerebellar ataxia (type 1, 2, 3, 6, 7, 8, 17); HD—Huntington’s disease; DRPLA— dentatorubral pallidoluysian atrophy; SBMA—spinobulbar muscular atrophy; CAG—cytosine, adenine, guanine.

This context prompts a closer examination on the normal and pathogenic CAG repeat ranges reported in the literature in recent decades, aiming to provide a clearer picture on the highly debated polyQ thresholds (in health and disease) and to correlate the trinucleotide repeat ranges with the most affected areas of the brain (illustrated in Figure 1).

**Table 1:** PolyQ disorders and their neuropathological aspects. Combined data from [115,116,117].

PolyQ Disease (Abbrev.)	Targeted Protein	Protein Size (kDa) *	Gene and Locus	WT Function of Protein	Normal CAG Repeats	Pathogenic CAG Repeats	Most Damaged Brain Areas	Neuropathology	Clinical Aspects
SCA 1	Ataxin-1	87	ATXN1, *6p23*	Chromatin binding factor; Transcriptional co-repressor	6–44	39–83	Cerebellum	Purkinje cells Dentate inferior olive Cranial nerve nuclei Substantia nigra Subthalamic nucleus Putamen Pallidum	→ Universal gait and limb ataxia → Dysarthria → Mild optic atrophy → Hypertonia (early)/hypotonia (late) → Dysphagia → Difficulties in breathing → Extrapyramidal findings
SCA 2	Ataxin-2	150	ATXN2, *12q24.1*	Stress granules; Possible participation in gene; Translational repression in vivo	15–31	34–200	Cerebellum	Purkinje cells Inferior olive Pontocerebellar nuclei Substantia nigra Striatum Demyelination of posterior columns Cerebral cortex Spinocerebellar tracts	→ Near universal gait and limb ataxia → Dysarthria → Abnormal eye movements → Neuropathy → Chorea → Dystonia → Dementia
SCA 3	Ataxin-3	48	ATXN3, *14q24.3-32.2*	Proteolytic processing of other proteins	12–41	55–84	Spinal cord	Globus pallidus Subthalamic nucleus Substantia nigra Dentate nucleus Pontine and cranial nerve nuclei Spinal neurons Peripheral neuropathy	→ Dystonia and rigidity (for type 1—early onset) → Cerebellar and pyramidal signs (for type 2) → Peripheral neuropathy (for type 3—late onset) → Parkinsonism (for type 4)
SCA 6	Ca^+2^ channel (α1A)	280	CACNA1, *19p13*	α1A-subunit of the voltage-dependent (P/Q) calcium channel	4–17	20–33	Cerebellum	Loss of Purkinje and granular cells Neuron death in dentate nucleus and inferior olive Atrophy in brain stem	→ Spasticity → Peripheral neuropathy → Dysphagia → Parkinsonism → Balance problems → Ophtalmoplegia
SCA 7	Ataxin-7	96	ATXN7, *3p14-21.1*	Regulation of gene transcription	4–35	37–306	Eye retina	Retina Cerebellar Purkinje and granule cells dentate Inferior olive Subthalamic nucleus and spinal motor neurons	→ Visual loss → Hearing impairment → Gait and limb ataxia → Dysarthria → Pyramidal findings → Dysphagia
SCA 8	CAG/CTG	40	ATXN8, *13q21*	Transcription and RNA processing	14–31	8–250	Cerebellum	Substantia nigra Loss of Purkinje neurons	→ Gait and limb ataxia → Dysarthria → Eye movement abnormalities → Extrapyramidal signs → Sensory neuropathy → Brain stem signs
SCA 17	Tata-binding protein (TBP)	42	TBP, *6p27*	General transcription initiation factor	25–42	45–63	Cerebellum	Small neurons in caudate nucleus and putamen Purkinje cells Thalamus Frontal and temporal cortex	→ Gait and limb ataxia → Dementia → Parkinsonism → Chorea/dystonia → Hyperreflexia
HD	Huntingtin	348	IT15, *4p16.3*	Cellular trafficking; Scaffold protein	6–35	36–250	Striatum	Cortex Basal ganglia Striatum	→ Involuntary choreic movements → Cognitive impairment → Dementia
SBMA	Androgen receptor	99	ARX-chromosome, *Xq11-q12*	Receptor	11–34	38–62	Spinal cord and brainstem	Anterior horn cells in spinal cord Dorsal ganglia	→ Muscle cramps → Walking disability → Decreased deep tendon reflexes → Dysarthria → Dysphagia
DRPLA	DRPLA protein or ATN1	190	ATN1DRPLA, *12p13*	Transcription co-regulator	3–35	49–88	Dentatorubral and pallidoluysian systems	Dentatorubral and pallidoluysian systems Basal ganglia Cerebellum	→ Ataxia → Choreathetosis → Dementia (adults) → Mental retardation (childhood) → Behavioral disturbances → Myoclonus → Epilepsy

* The protein masses are approximate, since variations in sizes depend on the CAG tract lengths.

For normal CAG repeats, the reported ranges in 2013 and then in 2020 were: 6–35 repeats for HD, 11–34 for SBMA, and 7–35 for DRPLA (see Table 2). For the latter two disorders, the pathogenic CAG repeats did not show significant differences from the scientific reports in 2013, to the ones from 2020. However, for HD, the reported pathogenic CAG repeats in 2013 were notably extended from ranges 36–170 to 36–250 CAGs reported between 2021 and 2022.

Regarding the normal ranges of CAG repeats in SCA disorders, the reported polyQ tracts fluctuated as follows. In 2013, the reported trinucleotide repeats were 27–36, and 6–35 in 2021 for SCA1. For SCA2, 15–32 CAGs were reported in 2013 and 2020, 17–29 repeats were reported in 2021, and then 14–31 in 2022. Other contrasting findings were distinguished for SCA3, where the reported CAGs ranging between 12 and 41 (in 2017 and 2020), in 2021, the range for wt conditions was 7–44 repeats. Similar numbers of CAGs were noted for SCA type 6 between 2013 and 2022, whereas different ranges were observed for SCA7 (7–35 repeats in 2013, and 4–19 CAG repeats in 2022). Identical polyQ tracts (of 25–42 repeats) were reported in 2020 and 2021 for SCA17, with slightly different ranges disclosed in 2013, 2017, and 2022.

A good agreement between CAG ranges reported in 2013, 2017 and 2020 was identified for pathogenic repeats in SCA1, SCA3, SCA6 and SCA17. Since the reported number of CAG repeats are based on multiple studies, each study involving particular context and experimental conditions, Table 2 provides just an overall perspective of the polyQ tract length’s evolution in CAG-related diseases. Here, longer polyQ tracts were noted within studies published between 2020 and 2022, when compared to those conducted until 2013, and 2017, respectively, particularly for pathogenic CAG repeats in HD, SCA2, and SCA7. 

The following section summarizes the essential aspects of each polyQ-related disorder, with a particular focus on full-length protein sequences, molecular hallmarks of abnormal polyQ aggregation behavior (e.g., autophagic impairment), and selective neuronal death. 

## 4. PolyQ Disorders

### 4.1. Spinocerebellar Ataxias

The CAG tract expansions in polyQ SCAs (SCA type 1, 2, 3, 6, 7, 8 and 17) are directly correlated with aggregation proneness and earlier (for longer CAG tracts) symptom onset, causing mitochondrial dysfunctions of cerebellar neurons, and other disruptions within the cellular homeostasis processes [127,128,129,130,131,132,133,134,135,136,137]. The most common form of SCA is SCA3, while the rarest type of this disease is SCA type 17. The SCA type 12 is not considered a polyQ-related disorder, despite the fact that SCA12 is also caused by extended CAG repeats—located in the untranslated region [138]. Nonetheless, the non-polyQ SCA disorders also involve altered disease-related protein functions and high toxicity patterns [130]. Further characteristics of polyQ SCAs are provided in Table 1.

Similar to other neurodegenerative disorders, in SCAs, the aberrant mitochondrial morphology and imbalances of oxidative–antioxidant system lead to severe dysfunctions in cell proliferation and differentiation processes [139,140,141,142,143]. As already known, the cellular homeostasis maintenance relies on balanced protein synthesis and degradation mechanisms, therefore in polyQ-related disorders, including SCAs, any alterations of particular cellular pathways amplifies the abnormal structural and dynamical patterns observed in neuronal degeneration [102,117]. Also, despite the protective effects of autophagy in synapse development and neuronal plasticity, by degrading the accumulations of abnormal proteins and/or dysfunctional organelles [144,145,146], there are also evidence suggesting that autophagic pathways might be a direct target for certain diseases, indicating that the accumulation of autophagic vesicles (AVs) is not only a result of increased autophagy, but might also be caused by decreased autophagic flux [102,146].

In terms of nuclear proteotoxicity in polyQ SCAs, there are two potential modes of actions based on: (i) increased nuclear retention and (ii) increased nuclear import of polyQ SCAs. for SCA-related proteins that originally reside in the nucleus [147], the accumulation of mutant proteins is presumably linked to faulty clearance of the multimerized toxic structures. Therefore, the ubiquitin–proteasome system (UPS) is the primary source of protein clearance in the nucleus, although a certain proportion of polyQ-related SCA proteins (in multimerized forms) might involve a slow or incomplete degradation by the UPS [148,149,150,151]. Moreover, both the UPS and autophagic pathways may decrease the quantity of nuclear multimerized forms of polyQ proteins, involving potential benefits against the toxic effects of extended polyQ tracts [147,152]. However, since translocation to the nucleus was not observed in SCA2 (not even for longer polyQ tracts), cytoplasmic clearance might be best suited for this type of SCA, where the toxic proteins accumulate in the cytoplasm. These findings imply that CAG repeat expansion alone is actually not sufficient for nuclear translocation [147].

On the other hand, data also suggest that overexpression of polyQ SCA3 proteins might perturb the function of UPS, while leading to abnormal increases in proteasome substrates. Consequently, longer polyQ tracts within SCA mutant proteins may also cause nuclear UPS impairment via seizing (in the cytoplasm) pivotal chaperons responsible for translocating cargo molecules to the nucleus [147,153]. 

Another important factor related to nuclear translocation is the proteolytic cleavage (previously discussed in Section 3). It is also known that, compared to full-length structures, cleaved proteins are rather toxic and more likely to initiate nuclear aggregates formation [97]. Since smaller mutant proteins are able to freely diffuse across the nuclear barrier (that separates the nucleus from the cytoplasm) and to accumulate in the nucleus, the size of polyQ SCA structures is of great importance. 

One explanation for the abnormal GOF linked to enlarged polyQ tracts is that polyQ expansions in proteins are linked to hidden signals for nuclear import, due to the existence of nuclear localization signal (NLS) sequences within protein structures. Another explanation, rather unlikely, is that unknown factors somehow recognize Q-expanded domains, facilitating the polyQ SCA proteins’ import. This possibility, however, comes in contradiction with the fact that polyQ SCA2 proteins remain in the cytoplasm although containing extended polyQ tracts. To conclude, a more plausible explanation here would be that, while alternating between the nucleus and cytoplasm (although primarily localized in the cytoplasm), SCA proteins with extended polyQ tracts become unable to pull out from the nucleus. Additionally, smaller multimerized SCA6 proteins might diffuse into the nucleus and become trapped, similar to SCA3 and SCA7 mutant proteins, due to abnormal interactions with nuclear proteins [147].

### 4.2. Huntington’s Disease

Huntington’s disease represents another progressive neurodegenerative disorder characterized by expanded CAG repeats within the HTT-exon1 sequence, ultimately being translated into enlarged polyglutamine tracts [154,155]. As a result of these extended polyQ tracts, mutant huntingtin (mHTT) proteins undergo atypical conformations and facilitate aggregations, leading to abnormal protein interacting networks or uncommon interactions with different cellular factors. The aberrant structural and dynamical behavior of mHTT proteins is based on two scenarios: (i) the H-bonded polar zippers and (ii) the transglutaminase-catalyzed cross-linking hypotheses [115,156,157]. 

The former hypothesis (i), also known as the polar zipper model proposed by Max F. Perutz [158] suggests that β-strands of poly-L-glutamine assemble into β-sheets or barrel configurations where strands are connected together via H-bonds between their main-chain and side chain amides. The latter hypothesis (ii) indicates that the aggregation behavior of mHTT structures is based on transglutaminase activity, and that the first-order rate constant of reaction increases for longer polyQ tracts (Q17-Q80) over a range of an order of magnitude, leading to increased cross-linking between mHTT and other interacting partners (including other mHTT proteins) [157].

**Figure 2:**
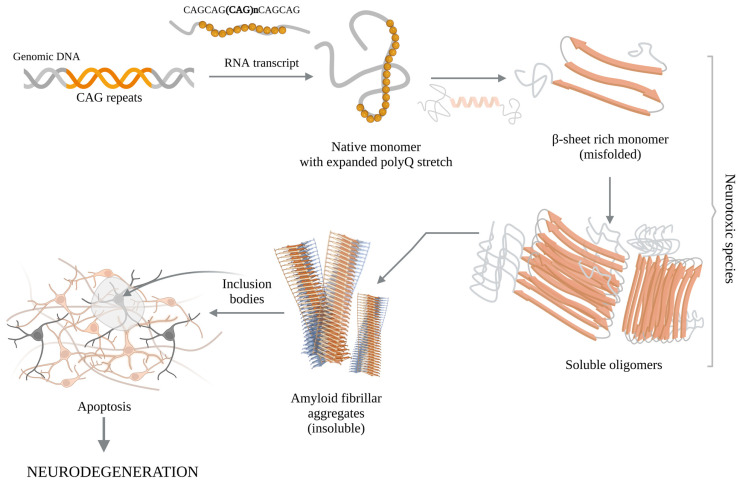
Molecular pathogenesis of polyQ diseases. Adapted from [80,88,169]. CAG repeats within DNA are translated into extended polyglutamine tracts. These tracts stabilize into β-sheets configurations, via transitioning from α-helical components, assembling into initially soluble β-sheet oligomers, which then transition into compact, highly stable and insoluble amyloid-like fibrils. These insoluble fibrils accumulate within neurons, inducing neuronal apoptosis and nervous tissue damages, triggering severe neurodegenerative effects. CAG—cytosine, adenine, guanine; DNA—deoxyribonucleic acid; RNA—ribonucleic acid; polyQ—polyglutamine.

In terms of protein structure, the HD gene (IT15) encodes a 3144 amino acid protein of 348 kDa. The full HTT sequence contains a total of 67 exons, where the polyQ tract (located at the level of the 1st exon) is preceded by the N-terminal domain consisting of 17 amino acids, and tailed by a proline-rich domain (PRD). For normal controls, the CAG repeats range between 9 and 36, whereas in pathological condition the CAG trinucleotides exceed the number of 36 repeats. Interestingly, in patients diagnosed with HD, the wild-type (wtHTT) and mHTT present similar distribution and expression pattern [115]. Moreover, although the molecular mechanism of polyQ pathogenesis (see Figure 2) has been first attributed to toxic GOF of mutant structures [159], the LOF processes of wtHTT were also seen to strongly contribute to HD progression [160,161]. 

While polyQ aggregates were initially considered to be toxic, several studies outlined the protective effects of large aggregates and suggested that smaller oligomers (containing polyQ stretches) represent in fact the toxic entity responsible for alterations in the protein folding landscape, mitochondrial disruption and autophagy impairment [124,162,163]. However, contrasting data indicate that polyQ oligomers/aggregates may not always correlate with toxicity, since in adult HD post-mortem tissues the degeneration of white matter within the caudate and putamen regions were not associated with polyQ aggregates. Cognately, for more aggressive forms of HD, the cerebellum subjected to substantial neurodegeneration lacks polyQ aggregates in post-mortem tissues, therefore implying that polyQ-independent toxicities might also impact the CAG-expansion processes related to polyQ disorders [84,164,165]. Just as important, the subcellular localization of these aggregates suggests a greater importance on toxicity assessment, rather than the length of polyQ stretches [115].

Another compelling aspect related to HD toxicity is that, even though it requires elevated polyQ concentrations, in vitro studies showed that normal ranges of CAG repeats can also form fibrillar aggregates. Nonetheless, proteins with a lower number of repeats impose much higher CAG concentration threshold, triggering a rather slower aggregation processes of HTT structures [166,167,168].

Huntington chorea is marked by increased muscle activity, impaired walking, and involuntary jerking, due to the fact that mHTT, despite its ubiquitous expression, aids neuronal degeneration in the striatum and—as the disease progresses—neuronal loss in the cerebral cortex [170,171]. Striatum degeneration has been correlated with the loss of medium spiny neurons (MSNs), the most abundant type of neurons within. Intriguingly, in HD conditions, the interneurons are considerably spared [172]. The interneurons act as intermediates between the afferent (sensory) neurons, receiving signals from the peripheral nervous system, and the efferent (motor) neurons that transmit signals from the brain. They also build connections to other interneurons, allowing for proper cell-to-cell communications. Therefore, the corticostriatal pathways’ disruptions were shown to be the cause of MSN degeneration. Additionally, other potential causes include the impairment of Na+/K+ ATPase, diminished mitochondrial complex (II, III) activity, and elevated dopamine concentrations (basal ganglia) [155]. 

An explicit reasoning behind the selective neuronal death in polyQ and other neurodegenerative diseases remains to be determined. For HD, a general assumption in this regard is that the preferential striatal degeneration and striosomal neuronal loss develop due to susceptibility, and not specificity. The structure-specific protein expression [173] and transcriptional dysregulation [174] are also considered as key contributors to neurodegeneration and neuronal vulnerability, particularly in HD. 

With regard to autophagy in polyQ disorders, Cortes and La Spada reviewed in 2015 the fundamental importance of the catabolic pathway required for CNS function and the maintenance of protein/organelle quality control in neurons. Their review paper, suggestively entitled “Autophagy in Polyglutamine Disease: Imposing Order on Disorder or Contributing to the Chaos?” [102] reminds us that polyQ structures are duplicitous, and “play a dual role” as both autophagy substrates and wrongdoers. In HD, the gross accumulation of AVs and alterations in the endo-lysosomal network are highly relevant for disease’s pathogenesis. However, when AVs were formed at normal rates in HD cells and were properly cleared by lysosomes, several studies showed that AVs failed to effectively entrap cytosolic cargo in their lumen [175,176,177]. Similar studies suggest that this behavior appears due to faulty recognition of organelles and/or aggregates by AVs [178,179,180]. Additionally, the loss of wtHTT proteins may compromise autophagic induction due to “empty autophagosomes” defects [181]. All these findings clearly suggest that HTT structures perform a large variety of autophagy-related functions (see references Cortes et al. [102] and Rui et al. [181]), including the selective recognition of cargos, autophagy induction and its neuroprotective role against this devastating brain disease. 

Another important aspect, in 2013, Rodríguez-Quiroga et al. [182] suggested that in some cases HD could have an atypical onset, indicating movement disorders like parkinsonism, tics, dystonia or ataxia. Also, on onset, HD can manifest identical symptoms with SCAs (e.g., SCA17), particularly for patients showing signs of dementia. Therefore, HD should be included within the differential diagnosis in patients with ataxias [182]. In agreement with this, in 2020, Franklin et al. [183] stated that cerebellar ataxias might be underestimated, and that the cerebellar involvement may significantly contribute in understanding the symptoms occurring in HD [183]. 

### 4.3. Spinal and Bulbar Muscular Atrophy

All polyQ diseases are autosomal dominantly inherited, except for SBMA X-linked neuromuscular disease, which is caused by polyQ expansions within the exon 1 of AR gene on chromosome Xq11–12 [116,184,185]. SBMA has a full disease penetrance restricted to adult males [178], is characterized by late onset and, as a unique feature, by both lower motor neuron and skeletal muscle pathology, including progressive muscle atrophy [186,187]. The neuropathogenesis stems from both loss and gain of function in the diseases-related protein with polyQ tract. However, as for the other polyQ disorders, the exact mechanism of how CAG repeat expansions in AR lead to motor neuron vulnerability and degeneration still remains indefinite [126].

The AR gene consists of eight exons, with the 1st exon encoding the amino-terminal transactivation domain (NTD) that contains the CAG repeat. Additionally to the polyQ tract, the N-terminal domain of the AR protein also contains polyglycine and polyproline (polyP) stretches. These stretches are not necessarily implicated in disease pathology, yet their functional roles remain poorly understood. The N-terminal fragment of AR is followed by a DNA-binding domain (DBD) allowing for specificity and stabilization of DNA–protein interactions, a hinge region—with a potential role in targeting proteins for degradation—and a carboxy-terminal ligand-binding domain (LBD) that undergoes ligand-dependent conformational changes and shows weak interactions with transcriptional co-regulators [187].

Although AR is a ligand-activated transcription factor, it also plays crucial roles in other cellular pathways. Upon binding to its ligand testosterone and entering the nucleus, polyQ-extended (>34 CAG repeats) AR proteins tend to misfold and to interfere with transcriptional co-activators, such as CREB-binding protein. Moreover, the expression of AR mutant protein in skeletal muscle seems to be a major driver of the disease, based on the presence of muscle fiber dysfunctions in patients with SBMA and experiments in mouse models. In terms of disease causation, the expanded polyQ tract changes the conformation of AR proteins from random coils to β-sheets, with particular alterations at their N-terminal fragment, triggering the neurodegenerative effects via a GOF mechanism [185,188]. Notably, the polyQ expansions in AR are also accountable for the partial loss of the protein’s normal function, by disrupting the interactions between NTD and transcriptional co-activators. Hence, similar to other degenerative proteinopathies, the proteotoxicities caused by polyQ tract expansions in SBMA occur via a hybrid complex of mechanisms mediated by protein unfolding/misfolding and the loss of AR protein’s normal function [187]. 

In good agreement with the existing hypothesis that oligomeric species are highly correlated with toxicity, the structural change from random coil to β-sheet configuration of the AR protein may favor the formation of soluble oligomers, considered to be rate-limiting in aggregation [189] and intrinsic toxic species that initiate a complex downstream series of events, leading all together to immunoreactive intranuclear inclusions’ formation and ultimately cell degeneration [190,191]. However, similarly to other polyQ diseases, in SBMA, these inclusions may also exhibit a protective role against pathogenesis by insulating the mutant proteins and hampering toxicity. 

Until 2011, several studies aimed to provide further insights into misfolded configurations and/or aggregated states of disease-related proteins, by developing antibodies that recognize specific secondary and tertiary structures, responsible for neurodegeneration [192,193,194,195]. Moreover, other reports [196,197] demonstrated that polyQ accumulations (in SBMA) in a diffusible form are more common, compared to nuclear inclusions in the anterior horn of the spinal cord. Also, since the relevance of mutant AR accumulations in cytoplasm of a certain type of cells remains rather uncertain, studies have confirmed that the nuclear translocation of polyQ AR proteins is required, but not sufficient for toxicity in model systems [187].

Studies also indicate that, mutant AR overexpression leads to accumulation of the marker protein involved in autophagosomes and autolysosomes’ formation (LC3), and the augmentation of dense AVs [191,198,199]. Therefore, modulating the transcription factor EB (TFEB) activity—involved in regulating the expression of lysosomal elements—could represent a reliable strategy for therapy development against SBMA [200], and other disorders characterized by defective autophagic flux [102]. Importantly, as in HD and other polyQ diseases, autophagy might also display a neuroprotective role in SBMA [102,199,201,202,203], while—in contrast—excessive activation of autophagy has also been found responsible for accentuating SBMA-related neurodegenerative phenotypes [204].

### 4.4. Dentatorubral Pallidoluysian Atrophy

As for the current understanding of DRPLA, this rare, autosomal dominant neurodegenerative disorder is characterized by ataxia, progressive myoclonic epilepsy, dementia, and psychiatric disturbances. The disease combines degeneration of the dentatorubral and pallidoluysian systems of the CNS, and may also involve damages of the cerebral white matter with diffuse myelin pallor, axonal transport impairment, and reactive astrogliosis. The unstable CAG repeat expansions at the level of the 5th exon of the atrophin-1 (ATN1) gene is the cause of DRPLA’s onset. Full penetrance occurs for polyQ tracts longer than 48 CAG repeats [205,206,207,208,209,210,211]. The human DRPLA gene consists of 10 exons. Also, DRPLA protein is localized (predominantly) in the nucleus and functions as a transcriptional co-regulator [205,212]. Similar to SBMA, stretches of polyserine and polyP exist near the CAG repeat tract of DRPLA protein. However, according to S. Tsuji and others, the lengths of polyserine and polyP stretches are not highly polymorphic [212].

Interestingly, studies indicated that mutant DRPLA structures are being expressed within similar levels to wild-type proteins. Consequently, it has been suggested that CAG expansions do not alter the transcription/translation efficiencies of the mutant gene, therefore toxicity, due to expanded CAG stretches, is more likely correlated with a GOF scenario. Moreover, the diffuse accumulation of mutant proteins in the neuronal nuclei seems to be the predominant pathological effect of DRPLA, and not—as suggested for other polyQ disorders—the formation of intranuclear inclusions. This type of (diffuse) accumulation involves regions of CNS beyond the reported affected areas [212]. 

The accumulation of AVs in DRPLA models, where truncated forms of ATN-1-75Q are being expressed, were correlated with decreased lysosomal function accompanied by intra-lumenal lysosomal lipofuscin and cytosolic accumulations. Thereupon, polyQ-ATN1 expressions can indeed block lysosomal degradation, but not lysosomal acidification or fusions with autophagosomes [213]. The exact mechanism of lysosomal clearance in DRPLA remains unknown, although one possible scenario suggested by Nisoli et al. is that autophagic processes may halt due to accumulations of partially digested autophagosomes. Certainly, this hypothesis outlining that the induction of autophagy increases neurotoxicity in DRPLA Drosophila models by accelerating the formation of giant autolysosomes with undigested contents [102,213], comes in contrast with other reported polyQ disorder-related autophagic impairment (highlighted in Table 3). 

The following table presents a comparative overview of polyQ disorders, highlighting both their differences and similarities. The distinctions of interest include inheritance patterns, diseases pathologies, genetic mechanisms, proteotocixity and affected CNS regions—based on the selective vulnerability of each disorder. Table 4 also focuses on shared traits, including molecular, genetic and functional similarities. 

## 5. PolyQ Accumulation Behavior and Theoretical Models

### 5.1. Aggregation Patterns

It has been demonstrated from both theoretical and experimental perspectives that extended polyQ tracts undoubtedly lead to neurodegenerative conditions. However, there are also evidence suggesting that for isolated polyQ peptides, regardless of their lengths, the structural properties and even the aggregation propensities do not exhibit significant differences [196]. Moreover, the hypothesis that monomeric structures depend sharply on polyQ length was refuted, suggesting that with the increase in polyQ domains, the resulted monomers present a rather collapsed, globular compaction [214], or random coil structures, as suggested by others [79,215]. 

Several in vitro studies have reported that the extended polyQ tracts undergo drastic structural transitions from native monomers into β-strand configurations, following further transitions from soluble oligomers into insoluble aggregates [80]. The functional implications here would be that those soluble oligomers are intermediate species consisting of β-sheet-rich molecules that reveal substantial neurotoxicities [195,216,217,218,219,220,221,222]. Also, the resulted insoluble aggregates are mainly found within amyloid fibrillar structures. Consequently, and in agreement with various in vivo studies, it seems that the simple expansion of polyQ stretches is enough to trigger neurological impairment and neurodegeneration. While the polyQs alone can indeed become toxic in vivo, it is important to keep in mind that in some cases the polyQ expansions or the formation of polyQ-positive aggregates alone are not sufficient to cause neuronal apoptosis [80,127,223], and that the flanking regions of the host proteins [224] and the biochemical properties of PPIs network are also critical to understanding the disease mechanism, and for determining the implications on the CNS’s selective vulnerability.

It has also been shown that cross-β-sheet fibrillizations involve intermediate helical configurations, demonstrated to partially contribute to the polyQ toxicity [80,225,226], and that within polyQ extended structures, these helices are being stabilized by intra-helical H-bonds mediated by the side chains of the constituent glutamine residues. A more detailed description of the structural transitions between α-helices and β-sheet configurations is provided in Section 5.2. Nevertheless, the full-protein-sequence context also represents a key factor in influencing the structural behavior of polyQs, with a decisive impact on the glutamine-rich proteins’ aggregation [79,227,228]. 

In 2021, Mier and Andrade-Navarro summarized the evidence related to polyQ sequence motifs and affirmed that polyQ regions may be found either in disordered phases (in unbound states), in helical configurations (emphasizing their interacting state), or in β-arrangements of aggregates [79]. However, with a particular focus on the relevance of polyQ peptides sequence context, to accurately compare the studies focused on synthetic polyQ structures with different polyQ stretches, and within distinctive host proteins, might represent a difficult task. 

**Figure 3:**
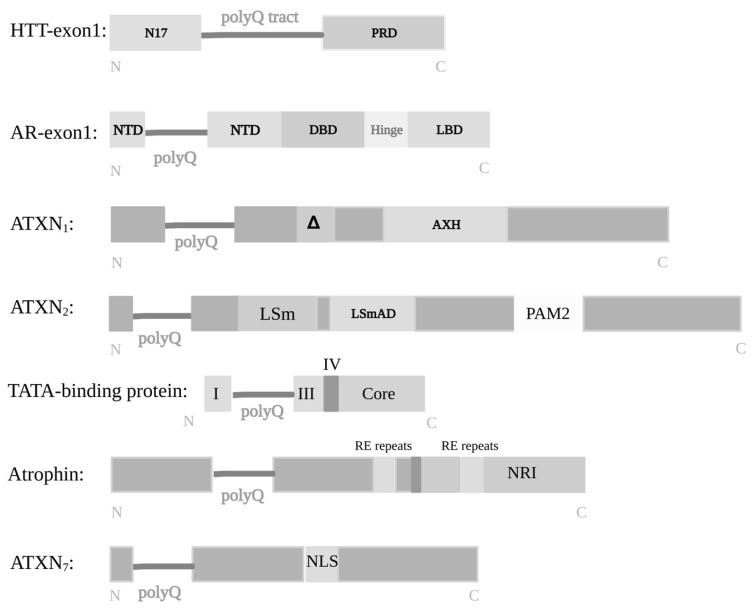
Diagram of host proteins in polyQ diseases, indicating the presence of polyQ tracts and their flanking regions. Adapted from Adegbuyiro et al. [236]. N—N-terminal domain, C—C-terminal domain, polyQ—polyglutamine, N17—N-terminal of HTT-exon1 with 17 amino acids, PRD—proline-rich domain, NTD—N-terminal domain of AR-exon1, DBD—DNA-binding domain, LBD—ligand binding domain, AXH—ataxin-1/HBP1 binding domain, LSm—N-terminal Like-Sm, LSmAD—LSm-associated domain, PAM2—Poly(A)-binding protein-interacting motif 2, Core—conserved region crucial in transcription initiation, RE—arginine-glutamic acid dipeptide repeats, NRI—nuclear receptor interacting domain, and NLS—nuclear localization signal motif.

What we know for sure is that within the host proteins there are no sequence or structural homologies, apart from the CAG’s abnormal repeats (Figure 3). Another common feature is the aggregation itself, highly dependent on the side chains of the polyQ tracts [229,230,231,232]. And ultimately, there is a general agreement that polyQ aggregates adopt fibril-like structures, indicating common features of antiparallel β-sheet structural components and fibrillar morphology [233,234]. Consequently, identifying the individual structural patterns of isolated glutamine stretches represents a more practical approach to understanding the properties of CAG repeats in aggregation, rather then analyzing the full length of polyQ-related proteins. It is also worth mentioning that these synthetic and isolated polyQ peptides might involve different aggregation patterns when compared to their aggregation in full-length structures [235]. 

### 5.2. Computational Studies

Theoretical approaches including molecular dynamics (MD), replica exchange MD (REMD) and coarse grain (CG) have been widely used in studying the polyQ aggregation pathways, and the large conformational transitions underpinning the polyQ-related proteins function. The main challenge of using these approaches is to resolve the sophisticated interplay of molecular properties whilst acquiring reliable statistical analysis of stochastic processes. These complex properties include, but are not limited to: monomeric sizes, chain lengths, protein contents, secondary structural components, gyration behavior, hydrophobic/hydrophilic profiles, and electrostatic interactions, all considered over specific environmental conditions. Given the primary focus of this review—to attain a comprehensive overview on the main challenging aspects, both theoretically (in this section) and experimentally (Section 6.1) of polyQ-related disorders—particular attention will be given to the most contrasting and recently published research papers.

As previously discussed, it is generally accepted that for different polyQ lengths in both monomeric and dimeric states, polyQ fragments may adopt β-sheet configurations with a high predisposition of forming aggregates. However, other structural components such as α-helices, β-sheets, poly-l-proline type II (PPII) helices and coils have been also reported in the literature [237]. The α-sheet structures, also known as α/polar pleated sheets, consist of similar H-bonding patterns as the β-sheets, with a distinctive orientation of the carbonyl and amino groups within the peptide bond units. Thus, since the carbonyl groups align (in the same direction) on one side of the pleat and the amino groups are disposed (also with the same orientation) on the opposite side of the sheet, one edge exposes the negatively charged carbonyls and the other side exposes the positively charged amino groups. Moreover, MD studies demonstrated that this type of structure may actually define the prefibrillar amyloidogenic intermediates in amyloid-related diseases [238]. Another structure of interest, less abundant than α-helices and β-arrangements but still highly correlated with fibrillar, folded/unfolded proteins, is the PPII helical component. PPII helices are defined by (φ,ψ) backbone dihedrals of roughly (−75°, 150°), are relatively open and have no internal H-bondings. Additionally, these types of helices do not necessarily contain repeating prolines, although proline residues present a high PPII propensity. In terms of function, PPII helices are involved in transcription and cell motility, with a dominant structural role in amyloidogenic proteins [239]. 

In this section, the integration of several key findings on the structural and dynamical behavior of polyQ stretches, based on theoretical studies published during the last decade, is prioritized. Emphasis is placed on the structural transitions from α-helices to β-sheet configurations, on solvent–polyQ interactions, interchain entanglements, parallel/antiparallel β-sheet stability, and the biophysics of polyQ aggregation mechanism.

The study conducted by Moradi et al. [240] demonstrated that, in agreement with the authors’ initial hypothesis, modulation of solvent–polyQ interactions might be used as a potential therapeutic strategy against polyQ disorders. Consistent with other CG studies, their MD results showed that in an explicit water environment, as the polyQ monomer tract’s length increases the polyQ’ solubility decreases. At the same time, the tendency for compact structures formation due to intra-polyQ H-bonds increases. Therefore, the aggregation rates turn higher for extended polyQ tracts, as expected, which ultimately correlates with reduced solvent–polyQ interactions.

The relevance of protein sequences with homopolymeric CAG expansions was largely addressed over the years using both theoretical and experimental approaches [79,117,227,228,241]. Noteworthy, the report of Ruff et al. predicts that interchain entanglements are diminished in the presence of amphipathic N17 stretches, via reductions in the frequency of intermolecular associations between globular polyQs. This finding suggests the possibility of designing specific sequences able to modulate the entanglement rates, and to investigate these modulation effects within in vitro aggregates [241].

The conformational pattern of polyQ segments with different chain lengths and the aggregation of peptides containing either just Qs or polyQ stretches with their corresponding flanking regions at the terminal positions were addressed by Baskar et al. [242] using both quantum mechanical (QM) and MD approaches. Their focus was mainly oriented towards regular secondary structure of polyQ stretches, chain length dependencies, the role of amide linkage in side chains, and structural stability of Ac-(Gln)n-NHMe peptides with variable polyQ chain lengths (1–16 Qs). As a result, the conformational behavior of polyQ peptide models was indeed chain length dependent, and governed by the interactions through peptide bonds of the side chain amide linkages. In addition, the QM predictions showed PPII configurations for some of the constituent Q residues. For all peptides, the most stable states were associated with C=O-C=O, CH-O and H-bond interactions [242].

In the same year, Chiang et al. [243] elucidated the structural stability of parallel or antiparallel β-sheet configurations. Performing all-atom REMD productions for systems consisting of one and two polyQ peptides, their findings showed that separated peptides tend to adopt helical configurations. The transitions from helix to β-sheet components emerge when the interpeptide distances become significantly shorter. Here, the unfolded states of helices promote higher propensities for intrapeptide β-sheet formation. However, the intrapeptide β-sheets are not necessarily considered as intermediate states between the helical configurations and interpeptide β-sheet transformations, since helical components can also turn into β-sheet structures without the presence of intrapeptide β-sheets. Noteworthy, random coil configurations were also observed along transitions from α-helices to β-sheets. Moreover, in agreement with previously proposed experimental models and other CG molecular dynamics studies, Chiang et al. differentiated the parallel and anti-parallel β-sheet structures and demonstrated that in interpeptide β-sheet assemble, the antiparallel β-sheets are more stable than the parallel β-sheet orientations [243].

Conflicting models suggested that polyQ-related structures predominantly endorse random coil configurations, facing contrasting perspectives on α/β-helical and β-sheet structural components [196,244,245,246,247]. This conformational context in PPI systems was extensively addressed over the last decade, emphasizing the polyQ-containing peptides’ aggregation mechanism and function. It has been shown that, depending on the polyQ threshold, the extended tracts might facilitate subsequent interactions with proteins containing longer and/or shorter polyQ stretches [248,249]. With reference to the potential effects of homorepeats flanking polyQ segments, it is now clear that polyP regions, often found in close proximity to polyQs, restrict the aggregation pathways of pathogenic polyQ stretches. Conversely, polyalanine (polyA) regions may initiate polyQ aggregations via α-helical coiled-coil formation [250,251,252]. In 2017, Totzeck et al. analyzed the structure–function correlations of the natural protein structure context of polyQs with different thresholds. Their conclusions indicate that, even for lower thresholds, the helical components are preferably N-terminally located to the polyQ middle position, while the random coils are oriented towards the C-terminal end. This structural pattern highlights that polyQ function is tightly associated with helical and random coil structural context, and that “even short stretches of repeats can serve this function” [89].

Detailed kinetic studies on polyQ aggregation mechanism revealed that elongated polyQ peptides aggregate via nucleated growth polymerization, and that over short repeat lengths, the size of critical nucleus increases from monomeric to dimeric and tetrameric states. It was initially reported that the fibril network may be responsible for amyloid-related diseases progression; however, recent theoretical data show that pathogenic phenotypes might actually derive from the toxic oligomers initiated through nucleation and growth processes [253]. It is also believed that polyQ aggregation pathways significantly vary from one peptide model to another, particularly due to variations in the critical nucleus sizes found for distinctive polyQ repeat lengths. The central parameters that characterize this two-step aggregation mechanism include the lag phase and the aggregation rate. Experimentally, these factors are difficult to be measured by virtue of the stochastic nature of the involved processes, and the increased heterogeneity of the resulted contents. On this basis, Haaga et al. aimed to provide a better understanding on the biophysical properties of polyQ aggregates, and found that, as reported by experimental data, the critical nucleus is polyQ tract length dependent. For clarity, the critical nucleus is defined as the number of monomeric units involved in an energetically unfavorable aggregation process (nucleus formation). Additionally, the nucleation barrier height decreased with the increase in Q-repeat lengths. While the models with 15 Qs presented a small nucleation barrier at the highest end of examined temperature range, models with 20 and 25 Qs revealed no nucleation barrier. Also, the nucleation time-lag increases for longer repeat tracts. In the same study, changes in oligomer composition showed β-sheet formations within short polyQ constructs and β-helical structures for longer polyQ tracts (e.g., Q30) [253]. 

Recent theoretical data [254] highlighted the relevance of vdW volumes in stability predictions, kinetics and thermodynamic behavior of aggregates. The aggregation tendencies of peptides/proteins are believed to be linked with decreased overall net charges, high hydrophobicity and elevated β-sheet propensities of the consisting amino acids. In globular proteins, the hydrophobic amino acids correlated with increased aggregation rates include Trp, Tyr and Phe residues. In general, the hydrophobic stretches are known to initiate aggregation processes. Compellingly, polyQ peptides are not only intrinsically disordered, but also hydrophilic. Moreover, in contrast with other theoretical models, the study conducted by Mishra and Thakur et al. show lower tendencies for β-sheet formations (during aggregation) in their polyQ peptide models. These tendencies were associated with Trp, Phe, Val and Ile amino acids. According to their findings, the residues’ hydrophilicity/hydrophobicity does not seem to play a critical role in the aggregation pathways of their mutant polyQ models with 46 residue length and interrupted by Pro-Gly residues within regular intervals. Additionally, the mutational analysis revealed that β-sheet contents of other globular/amyloid systems do not correspond to the polyQ’s β-sheet aggregation motifs [254]. As already mentioned, the overall charge of amyloid-prone proteins represents another key factor linked to their aggregation kinetics. It has been demonstrated that higher net charges on these structures are able to restrain self-association of their monomers, inhibiting aggregation and ultimately amyloid formation [255,256]. All things considered, it remains uncertain how exactly the amino acids’ hydrophobicity and their reduced net charges impact the polyQ aggregation mechanism. It is now clear, however, that specific mutations along these homopolymeric peptides may modulate their aggregation, and inhibit the polyQs’ associated toxicities [254]. 

Seamlessly aligned with potential strategies for disrupting extended polyQ peptides, in 2020, Moldovean and Chiş [257] explored the impact of specific key-point mutations along the helical conformation of the mutant HTT-Exon1 protein on the formation of toxic helical content related to HD. Using all-atom MD simulations, the authors investigated the structural and dynamical changes induced by these mutations, focusing on three sets of mutations where Q residues are mutated into proline residues. Their results showed that these mutations, especially when located at strategic points along the helix lead to greater structural transitions from (insoluble or hardly soluble) α-helices to highly soluble structural components like bends, turns, and random coils. Moreover, their findings highlighted that mutations in the middle and the edges of the helix had a greater impact on disrupting the toxic and compact helical structure. This suggests that replacing Q with proline in the polyQ tract could reduce the formation of helical structures, with no β-sheet formation observed, supporting the hypothesis that proline can disrupt both α-helix and β-sheet contents. The study proposes a threshold of nine proline residues for significant helical disruption rates and emphasizes the need for further dynamic investigations to understand the interactions of these novel mutant models with other cellular counterparts. A year later, the authors built on their previous work [257] by further exploring the interactions between mHTT models and calmodulin (CaM) [258]. This time, the authors investigated how their previously developed mHTT models, especially those with key-point mutations (such as the 9P(EM)), interact with CaM. The findings suggest that these mutant models show different binding affinities, total interaction energies and induce distinct structural changes within CaM’s structure [258]. Consequently, this research enhances our understanding of mHTT–CaM complexes and their implications in HD, offering insights into potential therapeutic strategies targeting these interactions. 

Noteworthy, another interesting MD study [259] showed promising perspectives towards the involvement of 2D-nanomaterial structures in extended (supercompact) polyQ tracts’ structural behavior (folding/unfolding). Feng and co-workers investigated two polyQ peptides, one consisting of 22 Qs and the other 46 Qs, modeled in interaction with both graphene and MoS2 nanosheets. Q22 presented similar binding modes—unfolds and elongates—on both graphene and MoS2 surfaces, however, regardless of Q22’s initial configuration. The initial (collapsed) configuration of Q46 showed no changes in its supercompact structure upon bindings to both nanosheets. A detailed analysis indicated that the differences between the two polyQ peptides’ behavior are mostly based on the competition between polyQ intra-H-bond interactions and the hydrophobic polyQ-nanosheet contacts [259].

Regarding the structural heterogeneity of polyQ-containing structures, Barrera et al. [260] conducted a cutting-edge CG study on homogeneous polyQ and heterogeneous Q-rich peptides aiming to investigate the Qs’ involvement in the early stages of aggregation. A notable finding here is that glutamine residues seem to serve a double purpose in seeding aggregation: first they initiate intermonomer contacts governed by hydrophobic effects, becoming afterwards the dominant mediator for (low molecular weight) oligomer associations. As expected, for heterogeneous systems the most relevant parameter is represented by the ratio between Q and non-Q amino acids. On the one hand, in Q-poor peptides, the glutamine residues remained unsusceptible to aggregation, outlining reduced interpeptide contacts due to electrostatic limitations. On the other hand, Q-rich peptides formed large and unstructured aggregates stabilized via internal Q-mediated interactions. 

Multiple possible polyQ structural models were designed and studied using MD techniques, in order to resolve the polyQ nucleation in the very early stages of aggregation within polyQ structures of distinctive sizes and shapes. In a study conducted by Zhou et al. [261], the initial configurations of interest were based on various β-helical models: left-handed circular, right-handed rectangular, and left- and right-handed triangular. The authors suggested that models consisting of both right-handed rectangular and left-handed triangular conformations are the most stable ones, and involve a helical structure of at least three rungs. Moreover, the nucleation seed for polyQ aggregation largely depends on how β-turns and β-sheets are preserved during these early stages. Hence, as previously stated within the literature, it is imperative to also investigate the effects of other secondary structural components on polyQ stability, since the formation of critical contacts between different structural elements may be the actual driving force for polyQ proteins folding and stabilization [261]. On this note, accumulating evidence clearly suggests the need for a better understanding of the structural heterogeneity in intrinsically disordered structures, and to potentially highlight the transitions that may lead to the formation of the pathogenic aggregates—under the form of aggregation landscapes in neurodegeneration.

Undeniably, the development of AlphaFold and RoseTTAFold algorithms ‘fueled the interest in protein structure prediction’, being a game changer in the study of protein (dys)function and the design of novel polypeptides with influential medical and/or industrial applications. In a nutshell, AlphaFold is an AI system that accurately predicts, through multisequence alignment, the 3D structure of a protein merely from its amino acid sequence [262,263,264,265]. Although this represents a major achievement in structural biology, we are still far from predicting and understanding the role of protein dynamics and the corresponding flexibility in protein function. One of the remaining challenges is to exceed the limits of single-structure prediction towards solving the proteins structural distribution (conformational landscapes), along with the meaningful atomic motions, that may be influenced by ‘external’ and ‘internal’ perturbations—due to and upon additional structural changes from one state to another. 

## 6. Structure–Dynamics–Function Correlations

### 6.1. Experimental Studies

As already known, the polyQ helices stabilization occurs via Q side chain to main chain H-bonds. These types of interactions have been observed for helical structures within the polyQ tracts of AR and HTT proteins. Recently, Escobedo et al. [266] demonstrated that polyQ helix–coil equilibrium is particularly sensitive to environmental conditions, and that the helices become highly stabilized at relatively low temperatures. In contrast, high solution temperatures favor polyQ coil configurations. The equilibrium helix–coil states are mainly governed by the N-terminal flanking region, solution conditions and tract lengths. The experimental studies focused on the structural traits of polyQ tracts, however, showed inconclusive results. For example, smFRET and NMR investigations showed little to no influence of tract length in HTT proteins [228,267], while other studies indicated that helical propensities of polyQ tracts found in AR and TBP increase upon CAG expansion [227,268]. 

The structural assemble of polyQ domains were also studied using fluorescence lifetime imaging microscopy detection of Förster resonance energy transfer (FLIM-FRET), emphasizing the great importance of the intramolecular proximity between the CAG tracts and flanking regions. Moreover, experimental studies accentuate on the relevance of additional cellular factors (environmental ones) with pivotal roles on deciding whether a polyQ tract, of a specific length, follows the toxic aggregation pathway or not. In comparison to the other polyQ-related disorders, the HD attracted most of the attention among the researcher, being in the spotlight of the polyQ extension experimental investigations. As a common ground shared among scientists, the polyQ repeat length positively correlates with the aggregates formation, and negatively with the HD onset [164,269,270,271,272]. Interestingly, recent studies showed that HD onset is actually based on the presence of uninterrupted CAG tracts, as opposed to the polyQ stretch hypothesis. The longer the CAG tracts, the frequent the aggregates. In terms of the cellular localization, which can be further correlated with neuronal vulnerabilities, the aggregates with longer CAG tracts are found in the nucleus, whereas for aggregates of shorter CAG tracts are found in the perinuclear space and cytoplasm. Also, while it is commonly acknowledged that CAG expansion in a single allele is enough for triggering HD, the interactions between wt and mHTT alleles may also boost disease progression and severity. Hence, polyQ expansion itself may not be the only determinant factor when it comes to HD, but the co-aggregation kinetics between wt-mHTT may serve as the cause of LOF scenarios for HTT proteins with shorter polyQ tracts as well [273,274,275,276]. 

The expression levels of polyQ-containing proteins are crucial parameters for assessing their solubility and cellular toxicity. However, it is important to keep in mind that the flanking regions of polyQ proteins are equally essential for aggregation and fiber formation. It has been demonstrated that the N-terminal domain of wtHTT is able to inhibit the aggregation of various proteins including α-synuclein [277,278,279]. Within this context, other studies have been also focused on the aggregation of HTT fragments with distinctive polyQ stretches. Indeed, the N-terminal fragment of wtHTT (e.g., with 25 Qs) may reduce fibrillation/aggregation by modifying the misfolding pathways of mHTT (e.g., with 51 Qs, 72 Qs, and 103 Qs) structures [280], and many other aggregation-prone proteins (e.g., α-synuclein, p53, and Rnq1). Furthermore, in vivo membrane interaction with HTT requires the N-terminal domain [176,281,282]. Worth mentioning, it has been confirmed that the N-terminal domain enhances polyQ oligomerization [176,281,283,284,285,286], being also involved in seeding and maturation processes [283,287]. Clearly, the N-terminal sequence of HTT and the polyQ tracts mutually impact each other, along with their coupled conformational properties [282].

Regarding the interactions between membranes and polyQ stretches, in 2021, Marquette and co-workers used circular dichroism (CD) spectroscopy, Thioflavin T (ThT) fluorescence, and dynamic light scattering (DLS) measurements for a detailed investigation of the role of membrane in polyQ association kinetics. Their constructs consisted of membrane-anchoring HTT N-terminal domain followed by polyQ tracts of different lengths (9 Qs, 12 Qs and 17 Qs). As a result, HTT-polyQ membrane associations increasingly accelerates polypeptide aggregation rates. In addition, in the presence of membranes, peptides aggregate in a peptide-to-lipid ratio-dependent manner. For shorter polyQ fragments, peptides aggregate significantly slower, whereas the aggregation kinetics accelerates with the increasing number of Q residues [248,282,288,289,290,291,292,293,294,295,296]. 

Using CD, NMR and FTIR spectroscopy, Siu et al. [235] established potential therapeutic approaches targeting molecular mechanisms in polyQ diseases. The authors found that the insertion of Qs impacts the backbone conformation of host peptides, leading to destabilization of the β-hairpin structure and favoring oligomerization at higher concentrations. The kinetics underlying polyQ expansions usually follows: the expanded stretches undergo drastic conformational changes from their native states into β-sheet monomers, then the soluble oligomers are being formed, followed by the initiation of the insoluble aggregates. The soluble species (the β-sheet-rich monomers and oligomers) are the intermediate structures posing a great threat to the living cells [80,195,222]. In the cytoplasm, the extended polyQ-related mutants may exist in various forms: as soluble monomers and/or oligomers, and as insoluble aggregates—the well-known inclusion bodies. Importantly, the α-helical coiled-coil structures also show great contribution to the increased toxicity patterns of polyQ proteins [225,226]. Nonetheless, the sole existence of these polyQ aggregates does not necessarily correlate with cell death. Moreover, the experimentalists demonstrated that the formation of these aggregates may actually serve as a protective response of the neuronal cells against the soluble, intermediate, and toxic polyQ species [80,223]. Hence, all these findings show the great importance of conformational stabilization and aggregation inhibition of these polyQ expanded intermediate structures. In this regard, in 2021, Minakawa et al. [80] thoroughly reviewed the protein aggregation inhibitors as disease-modifying therapies for polyQ disorders. 

Significant efforts have been made in the recent years in order to understand the basis of regional, and therefore selective, neuronal vulnerability particularly through the development of mouse models that can imitate the spatial–temporal traits of polyQ pathology. Moreover, scientists have tried to identify and characterize the biochemical, morphological, and electrophysiological properties of the exposed neurons involved in these degenerative patterns. However, in spite of the monotonous and monogenic features of polyQ disorders, determining the exact mechanisms underpinning the selective neurodegeneration remains somewhat puzzling and awfully challenging. Why? Mainly because the functional aspects of most polyQ-related proteins are still unknown. 

Being a well-characterized transcription initiation factor, TBP is the exception which makes SCA17 an ideal disease model for selective SCA neuropathological studies. Liu et al. [297] used SCA17 mice for investigating the selective effects of mutant TBP. The overexpressed mutant TBP with 68 Qs and 105 Qs in different brain regions of wt mice traced the Purkinje cells in the cerebellum as being the most vulnerable region. Moreover, selective neuronal loss was also caused by the endogenously expressed mutant TBP (in SCA17 knock-in mice). The immunofluorescent staining and Western blotting results showed a preferentially and drastic loss of Purkinje cells in SCA17 knock-in models, when compared to the control ones. In agreement with the selective neuronal loss in SCA17 patients, their findings showed striatal pathology (characterized by reduced levels of DARPP32 protein) in 5-month-old SCA17 knock-in mice. Intriguingly, they also found that the overexpressed TBP with 44 Qs caused Purkinje cell degeneration to a similar extent as the overexpressed TBP with 68 Qs and 105 Qs, although without promoting the formation of aggregates in the cerebellum. The authors’ explanation in this regard was that ‘aggregated mutant TBP is unlikely to be as effective as soluble mutant TBP to bind transcription factors and to affect gene expression’ [297].

Other experimental approaches for investigating and finding a potential cure against polyQ-related disorders include the activation of protein degradation systems. As previously mentioned in this review, an essential protein clearance mechanism is represented by the autophagic processes. For example, the LC3B and/or SQSTM1 autophagic markers have been observed in polyQ aggregates and were correlated with autophagic impairment. Also, in the SCA2 condition, the levels of WDFY3 autophagic protein are higher when compared to the wt condition. However, it has been shown that autophagy activation/upregulation via chemical derivatives (in vitro and in vivo) significantly reduces the neuropathological effects in SCA3 mouse models, and mutant ataxin-2 aggregates in SCA2 models [298]. 

All these considered, the review paper of Jain M. et al. [299] specifically focuses on the role of autophagy, via highlighting the potential of targeting autophagy pathways as a therapeutic strategy. This approach includes modulation of autophagic activity to manage the accumulation and toxicity of protein aggregates, while offering a promising avenue for treatment development in these neurodegenerative disorders. Additionally, the therapeutic effects and modes of action of the available autophagic modulators in polyQ diseases are also provided. Nevertheless, further understanding of autophagy pathways is required, which may lead to multitarget approaches against toxic polyQ strands and neuronal degeneration—with the potential of significantly delaying diseases progression [299]. 

From a structural perspective, it has been clearly observed that coiled-coil regions are motifs for PPIs between polyQ-containing structures. Upon these interactions, it seems that flexible polyQ sequences tend to adopt highly structured helical configurations, thereby enhancing the strength of PPIs. This enhancement may lead to dysfunctional interactions, when polyQ regions are excessively expanded [79,121,225,300]. However, experimental data show that this behavior may be avoided, e.g., via disrupting the excessive CAG repeats with specific CAA mutations [90].

Although there are currently no disease-modifying therapies against polyQ disorders, significant research efforts are oriented towards developing treatments that might slow down the progression of these diseases, and that may alleviate particular symptoms. The most widely used method in modeling and research of polyQ disorders is CRISPR-Cas9 technology that has been employed for modifying the CAG tract in various cellular models: HEK293 cells, human fibroblasts, human neural progenitor cells (NPCs), HD patient-derived induced pluripotent stem cells (iPSCs), mesenchymal stem cells (MSCs), and BacHD transgenic mouse model [301,302,303,304]. In 2022, Karwacka et al. [125] thoroughly reviewed the advancement of gene-editing technologies that have undeniably impacted the polyQ-related disease research. The emerging treatment modalities for SCAs were also recently reviewed by Ghanekar et al. [134].

Lowering the levels of proteins containing expanded polyQ tracts shows great promise as a therapeutic approach against polyQ diseases. Therefore, extensive research has also been conducted on the use of antisense oligonucleotides (ASOs) and RNA interference in order to silence the genes responsible for the diseases’ onset [82,305,306,307,308]. For instance, a phase 1/2a clinical trial for HD treatment indicated that repeated administration of an ASO called tominersen directly into the cerebrospinal fluid (CSF) successfully reduced the levels of the mHTT protein [309]. In contrast, a phase 3 trial of tominersen was ceased because it did not show superior clinical effectiveness compared to a placebo [82,310,311]. One possible explanation for this is that the ASO’s concurrent suppression of the wtHTT gene could have compromised its normal function, while another reason could be based on the fact that tominersen may not have effectively reached the affected brain regions. Equally possible, a third reason might be based on the fact that ASO administration occurred too late, considering the accelerated HD’s progression, to improve its neurological symptoms. 

Another notable development in HD treatment comes with the experimental therapy WVE-003, studied in a phase 1/2 clinical trial (NCT05032196). The ASO is designed to selectively target and reduce only the m-HTT structures, while preserving the expression of wtHTT proteins. Single doses (30 or 60 mg) of WVE-003 administration appear generally safe and well-tolerated by HD diagnosed patients [82]. Other pre-clinical studies involve using zinc finger protein transcription factors delivered via adeno-associated virus (AAV) that specifically target and suppress the expression of mHTT mRNA (containing extended CAG stretches). Another promising candidate is represented by a divalent small interfering RNA (siRNA) molecule (consisting of two chemically modified siRNAs connected via a linker). Its intracerebroventricular (ICV) administration exhibited sustained silencing of the HTT gene in mice (effects lasted for at least six months) and non-human primates (effects lasted for one month) [312,313]. Nevertheless, it is imperative to note that studies conducted in pre-clinical models require further research for determining their safety and effectiveness in human clinical trials. Since reviewing the nucleic acid-based therapeutic approaches in polyQ disorders is slightly out of the scope in this paper, further insights into novel therapeutic candidates—for SBMA as well—can be found in the recently published paper by Hirunagi et al. [82].

In a study conducted by Schuster et al., the authors aimed to map the transcriptional changes across early, mid, and late stages of SCA3 disease in two selectively vulnerable brain regions: the cerebellum and brainstem. For that purpose, they used male and female age-matched transgenic mice expressing full-length (human) mutant ataxin-3 (ATXN3). Their findings suggested that the dysfunction in oligodendrocyte maturation is a result of a toxic gain-of-function mechanism specifically related to SCA3. Further investigations demonstrated a significant reduction in mature oligodendrocytes in brain regions susceptible to SCA3-related damage, and abnormalities in the myelination of axons—indicating disruptions within the protective coating around nerve fibers [314]. 

Thus far, mouse models have proven indispensable in unraveling the mechanisms underlying the development of SCA1 in the cerebellum [135]. Most of the pathological studies have been mainly focused, however, on the primary fissure and lobules V and VI, relying on bulk RNA expression or protein analysis [315,316]. Since the cerebellar Purkinje cells are mostly affected in SCA1 patients, despite the wide expression of mutant ataxin-1 (ATXN1) in the brain, researchers have also experimentally addressed the unresolved causes of the selective vulnerability for specific brain regions. For instance, Hamel et al. used ATXN1154Q/2Q SCA1 knock-in mice that express the mutant protein under the endogenous promoter, allowing for physiological levels and spatial distribution of mutant ATXN1 expression. By employing this model, they investigated the intracerebellar regional differences in pathology and the underlying molecular factors contributing to the disease [317]. Their findings revealed increased dendritic atrophy and loss of synapses, along with elevated reactive gliosis (its presence is not specific for a particular pathologic entity) in these regions. These observations shed light on the distinct pathological features in different cerebellar regions, providing valuable insights into the SCA1 progression.

The correlations between intracerebellar regional differences in pathology and ion channels’ role in neuronal excitability is a subject of significant interest in polyQ disorders’ research. Studies have indicated that the abnormal expansion of polyQ stretches in certain proteins, can lead to disruptions in the ion channel function, which in turn, affects neuronal excitability. These channels play a crucial role in regulating the ions flow (e.g., Ca^2+^, Na^+^, K^+^ and Cl^−^) across the cell membrane, and are therefore essential for maintaining a proper functioning of the nervous system. The intracerebellar regional differences in disease pathology may also be related to the varying expression levels and/or distribution of specific ion channels in these regions, while triggering changes in neuronal excitability, and altering firing patterns between neurons. Equally important, dysregulations of ion channels can also contribute to the aggregation and accumulation of toxic protein aggregates, further exacerbating neuronal dysfunction and neurodegeneration in polyQ diseases. A comprehensive overview on the biophysics of ion channels, and their involvement in SCAs, HD, SBMA and DRPLA, was outlined in-depth by Martinez-Rojas et al. [318]. It is imperative to consider that investigating the changes in neuronal activity within polyQ diseases, presents significant challenges. Researchers in this field aim to discover novel therapeutic possibilities, necessitating the development of innovative models and the adoption of multidisciplinary approaches to address these complex and difficult-to-treat conditions [318].

Another study that highlighted the complexity of mechanisms driving cellular vulnerability in disease, with a particular focus on SCA1, is the study conducted by Coffin et al. [319]. Likewise other polyQ disorders, while the mutation is present in all cells, only selected populations of cells degenerate in SCA1. It has been shown that interactions between the mutated ATXN1 and the transcriptional repressor CIC (a critical regulator of neuronal differentiation) are implicated in cerebellar Purkinje cell pathogenesis. The relevance of these interactions in other cells, however, remains uncertain. Upon mutating the ATXN1 gene in ATXN1154Q/2Q mice to prevent the ATXN1–CIC interaction, genome-wide CIC binding was normalized, but it only partially corrected transcriptional and behavioral phenotypes. Moreover, using unbiased proteomics, the authors found that three other ATXN1-interacting transcription factors (RFX1, ZBTB5, and ZKSCAN1) are potentially involved in disease pathogenesis as well [319]. 

With respect to SBMA, the gain-of-function-based toxicity was proven to involve phosphorylation processes, according to a recent investigation in the context of SBMA using HEK293T and HeLa cell lines, and mice models [320]. The study identifies that cyclin-dependent kinases (CDKs) and a Ca^2+^-dependent phosphatase, calcineurin (CaN), have opposing effects on the phosphorylation and function of polyQ-expanded AR. CDKs enhance the phosphorylation and toxicity of polyQ-expanded AR, while CaN reduces these effects. This discovery opens avenues for targeting these pathways, offering a promising therapeutic strategy against SBMA and similar neurodegenerative diseases [320]. 

With a particular focus on HD, Pigazzini et al. emphasized the increase in aggregation propensity in vitro, facilitated by the PRD. Importantly, this tendency for heightened aggregation was not observed in vivo; instead, mHTT-exon1 with an expanded polyP domain remained soluble over time. To examine the role of flanking domains within a physiological and cellular framework, the authors created the first Caenorhabditis elegans (*C. elegans*) strains expressing mHTT-exon1 neuronally. Using FLIM, they differentiated between the formation of soluble, oligomeric, and aggregated species in vivo, correlating these forms with toxicity. The findings highlighted the advantages of having an expanded polyP domain alongside an expanded polyQ sequence, by showing an in vivo solubility maintenance of mHTT-exon1 with elongated prolines—a beneficial trait against toxicity observed only for pathogenic Q stretches [321]. A similar behavior, by using all-atom MD methods, was also predicted in 2020 by Moldovean and Chiş [154] focused on how proline mutations alter the structure of mHTT-exon1 peptides. 

Due to its cyclic nature, proline is the only amino acid with detectable *cis/trans* isomeric equilibrium. This property within polyP tracts was uncertain until recently. Long polyP sequences exhibit reduced *cis* population for inner prolines, primarily at ≈2%, while termini prolines have ≈10% *cis* population. The stiffness of polyP is associated with its protective role in aggregation-prone polyQ regions. While PRD was also proposed to have chaperoning effects, so far in the context of HD, Zhang et al. [322] and others found a tight correlation between polyQ’s length and the primary polyP stretch—with an optimal polyQ:polyP length ratio of 2:1 [228,250,323]. Together, these studies highlight the significant influence of proline residues on the structural behavior of host protein associated with polyQ disorders. 

In 2022, the study conducted by Kapadia et al. [324] involved the development of small molecules able to disrupt the abnormal binding of mHTT protein to CaM (a key regulator in Ca^2+^ signaling). The authors used high-throughput in vitro and cell-based screening assays to identify compounds owning protective effects. Through iterative optimization, they developed specialized compounds effective in shielding against the cytotoxic effects of mHTT protein and normalizing intracellular Ca^2+^ release in PC12 cell models of HD. Their findings suggest that disrupting the binding of mHTT to CaM could be a promising approach for developing therapeutics for HD [324]. It is worth emphasizing the correlations between this particular study and the all-atom study focused on mHTT–CaM interactions [258], previously described in Section 5.2. Although the methodologies differ, computational simulations versus experimental observation, the core focus remains consistent: understanding the intricate dynamics of mHTT and identifying potential means of ‘breaking’ mHTT–CaM bindings (either via additional mHTT mutations [258] or via the development of small molecular disruptors [324]), known for their protective effects against polyQ-related cytotoxicity. 

### 6.2. Refined Models: Synergies between Theory and Experiment

In 2008, Truant et al. delved into the intracellular dynamics of mHTT protein, crucial to understanding the disease’s progression and potential intervention points [325]. By 2008, it had become evident that the initial theories proposing misfolded, precipitated polyQ proteins as a common pathogenic mechanism were being challenged by emerging research on polyQ diseases. Worth remembering, previous—but also subsequent—findings indicate that the harmful forms of protein in these conditions might be due to its soluble mutant conformers, emphasizing the significance of the protein environment surrounding the expanded polyQ tracts in determining disease specificity. This contradicts the earlier toxic aggregate hypothesis. For instance, in SCA17, it has been shown that even with over 40 CAG repeats, the TATA box-binding protein’s normal polyQ tracts do not always cause disease, and their expression does not universally harm cells. However, only manifesting as ataxia when polyQ stretches are expanded beyond 60 repeats [325,326]. 

Additionally, for HD condition, the pathogenic mechanisms involved do not delineate disease’s specificity in specific neuronal populations. A reasoning behind this would be that the term ‘polyQ’ is frequently misused for mHTT-exon1, and *aggregates* are broadly used for any polyQ-related protein clusters, regardless of their actual structure. This confusion extends to the use of huntingtin for only the exon 1 fragment, leading to mistaken beliefs about its properties compared to the full-length protein in HD. A key challenge here is determining if the pathology in HD is indeed fully represented by solely the exon 1 fragment of huntingtin, while ignoring the potential—and essential—role of the remaining 97% of the protein [325]. To succinctly summarize, the key to developing effective treatments for polyQ disorders likely lies in targeting the specific functions of the host proteins. This approach may prove more successful than solely focusing on polyQ tracts and their aggregation, as the surrounding protein context plays a critical role in mediating toxicity. These features were also explored and confirmed for diseases other than HD, such as SBMA and SCAs. 

Years later, Punihaole et al. complement previous structural studies based on MD/REMD simulations, by offering insights into the conformational states and transitions of polyQ peptides, particularly emphasizing the role of monomeric solution states in fibril formation [327]. Their study employed both experimental and computational techniques, while investigating the solution-state structures and conformational energy landscape of the peptide system D2Q10K2 (Q10) through Ultra-Violet Resonance Raman (UVRR) spectroscopy and MD simulations, building on their prior research. They discovered two distinct monomeric states of Q10 in aqueous solution (a collapsed β-strand-like structure prone to forming amyloid-like fibrils and a PPII-like structure resistant to aggregation) that do not easily convert into each other due to a high-energy barrier, as explored through metadynamics analysis. Their theoretical data aligned well with CD and UVRR data. Moreover, they explored on how low H-bonding and dielectric environments influenced Q10’s structure, observing the transformation of PPII-like conformations into α-helix and turn components—in acetonitrile–water mixtures, and the formation of β-sheet fibrils aggregates at high acetonitrile concentrations. Interestingly, fibrils formed in high acetonitrile environments dissolved back in water-rich solutions [327]. Additionally, their study offered contrasting and complementing findings from Wang et al. [328], who hypothesized that the structural disorder in polyQ peptides arises due to various potential intramolecular and intermolecular amide H-bonds. Wang et al. suggested that this complexity leads to disrupted formations of organized structures like β-sheets, fostering disorder [328]. The fact that Q10 peptide has a conformational energy landscape filled with numerous shallow energy minima and a variety of H-bonding interactions comes in agreement with Wang et al’s notion of a complex bonding pattern. However, Punihaole et al. also discovered well-defined structural states for Q10, being in contradiction to Wang et al.’s findings. Particularly, in the PPII-rich state of Q10, Punihaole et al. noted a predominant H-bonding with water. Based on these contradictions, the authors [327] emphasized that the complex energy landscape of polyQ peptides, filled with many local energy wells, might not be fully explored in classical MD simulations, potentially leading to Wang et al.’s [328] different observations. Punihaole et al.’s metadynamics approach, in contrast, samples a broader range of structures, uncovering global energy minima. Moreover, the choice of force field and water model significantly influenced the simulation results. In the latter study, the authors used the TIP3P water model and the CHARMM36 force field which differs from Wang et al.’s methodology, leading to different observed H-bonding interactions.

Buchanan et al. [234] and Escobedo et al. [268] continued along this line, examining protein structure and aggregation patterns in polyQ diseases. While Buchanan et al. provided detailed insights into the fibril structures of polyQ sequences, Escobedo et al. offered a broader perspective on the helical nature of polyQ tracts and their implications in polyQ pathology. Both studies relied on the synergistic advantages of the theoretical-experimental interplay, and while Buchanan et al. [234] emphasized the structural analysis of amyloid fibrils using 2D-IR spectroscopy and MD simulations, revealing that polyQ fibrils are indeed predominantly composed of antiparallel β-sheets (for Q24) forming a β-turn structured, Escobedo et al. [268] explored the helical nature of the polyQ tract in the AR—associated with SBMA (using QM/MM and MD calculations, along with CD and NMR)—revealing that helicity increases with tract length and is stabilized via unconventional H-bonds. The latter study provides insights into the correlation between polyQ tract length and the aggregation propensity in SBMA.

Relying on MD simulations, CD, NMR, and in vitro studies, in 2019, Hong et al. [329] highlighted how specific protein regions influence aggregation in SCAs. In contrast to other studies, mainly focused on general polyQ sequences, the authors highlighted the significance of the alanine-rich regions (ARR) in α-helices within the polyQ tract and their role in suppressing aggregation, a novel insight that was not covered before. Within their focus on ataxin-7 (Atx7), the authors’ demonstrated, on the one hand, that polyQ expansion tends to increase the aggregation of Atx7-N, and this coincides with the formation of stable (marginally) α-helices that become more pronounced with the polyQ expansion. On the other hand, the study reveals that ARR within Atx7-N initiates the formation of local α-helices in the polyQ tracts. Interestingly, these particular α-helices, especially within ARR2 motifs, play a significant role in inhibiting the aggregation of Atx7-N, even when the polyQ is expanded [329]. This highlights a complex interplay where certain structural elements both contribute to and mitigate aggregation, depending on their nature and the context within the host proteins.

Noteworthy, the recent study conducted by Kandola et al. addresses a critical aspect of polyQ diseases, focusing on identifying the essential features of polyQ amyloid nucleus. The research presents novel findings on the nucleation of pathologically expanded polyQs, involving segments of three Q residues. Using molecular simulations, DamFRET, SDD-AGE and amyloid predictions, the authors revealed a unique four-stranded steric zipper pattern that plays a key role in the aggregation process. The findings also highlight how this pattern can self-poison its own growth ‘by engaging naive polypeptides on orthogonal faces in a fashion characteristic of polymer crystals with intramolecular nuclei’, offering insights into potential therapeutic interventions for polyQ diseases [330]. Additionally, this research adds a new dimension to our understanding of polyQ’s molecular etiology, complementing the findings from earlier studies. While earlier research primarily focused on the cellular and molecular dynamics of mutant proteins involved in these diseases, Kandola and coworkers bring a more detailed structural perspective, particularly on the initial events leading to polyQ-related protein aggregation.

Beyond doubt, ML methods are increasingly advancing towards a new era in research, including within the context of polyQ disorders. These advanced computational techniques, when integrated with experimental data, can significantly enhance the accuracy of disease predictions, paving the way for the development of effective therapeutics against polyQ diseases. A prime example of this progressive approach is the study published by Hatano et al. [331] in 2023, which leverages ML for predicting polyQ diseases’ onset, illustrating the potential of combining computational predictors with experimental data. In their study, Hatano and coworkers compared the performance of two ML models, Random Survival Forest (RSF) and DeepSurv, with six conventional methods of parametric survival analysis. They aimed to predict the age-specific probability of SCA3 and DRPLA’s onset using survival curve analysis. The cross-validation and evaluation criteria such as the root mean squared error (RMSE), the mean absolute error (MAE), and the integrated Brier score were used. Amid the models investigated, RSF and DeepSurv outperformed the parametric survival analysis methods in terms of prediction accuracy. For both SCA3 and DRPLA, RSF exhibited superior accuracy compared to DeepSurv, as indicated by RMSE, MAE, and integrated Brier scores. Hence, by using RSF, the authors established age-specific probability distributions for age at onset based on CAG repeat size and current age. Hatano et al. also stated that one constraint of their approach was correlated with the limited sample size, which might have contributed to observed inversions in the predicted onset ages (the small number of cases may have introduced bias in the underlying data) [331].

Taken together, these papers (among others) underscore the importance of a holistic approach in understanding and counteracting polyQ diseases, integrating atomistic, molecular, cellular, and therapeutic approaches. Overall, the study of polyQ regions in proteins has largely been elucidated through NMR studies, supported by techniques like SAXS and CD, often in conjunction with computational methods (MD/REMD, CG, MC). For instance, by combining NMR and SAXS data with CG simulations, recent research on full-length ataxin-3—comparing normal and expanded polyQ tract lengths—revealed structural details of its flexible tail, which includes the polyQ repeat and ubiquitin-interacting motifs. This tail, despite its flexibility, tends to adopt a partially structured, extended conformation with short-lived collapsed conformations in specific regions. Additional evidence emphasized that areas adjacent to the polyQ tract in certain proteins influence the helical structure of the polyQ itself [8]. Generally, polyQ repeats are found to preferentially form α-helical secondary structures in experimentally determined 3D structures. However, the exact structural characteristics of polyQ regions, as identified in the Protein Data Bank (PDB) [332] via X-ray crystallography, NMR, or cryo-EM, present a diverse array. For instance, short polyQ sequences (5–7 Qs) may exist under various structural forms:

- As part of a loop linking two β-strands;
- Within the core of an extended, stable helix;
- At the terminal end of a helix, exhibiting some loss of helical integrity;
- As a segment of a shorter helix;
- At the onset of a lengthy, stable helix;
- Or within coiled-coil structures, maintained by polyQ-induced interactions between helices.

In contrast, longer polyQ sequences (9–14 Qs) are typically found at the end of elongated helices. Nonetheless, detailed structural data for regions longer than 15 Qs remain unavailable [8].

While widely recognized and familiar to the scientific community, PDB (https://www.rcsb.org/, accessed on 1 January 2024) continues to be a foundational element in bridging theoretical and experimental research. This database is more than just a repository; it is a gateway to melding experimental findings with the realm of computational methods. It enables researchers to predict and explore how polyQ lengths and contexts shape host proteins’ behavior. Moreover, using structural data as input systems, it enables us to design and model new variants of polyQ regions. The new models can then be further tested and validated through experimental methods. Last but not least, within the generated theory–experiment feedback loop, PDB provides invaluable data that may be used in pioneering drug design strategies, targeting the intricate puzzles represented by polyQ-related pathologies.

Alternatively, as an innovative resource—the PolyQ Database (https://polyq.pt/, accessed on 1 January 2024)—developed by Estevam et al. [333] is an integrated platform focusing solely on polyQ diseases. It was recently developed, and integrates vital information relevant to a wide audience: scientists, clinicians, and the general public. It encompasses a broad range of disease-related topics such as epidemiology, details about genes and proteins causing these disorders, their pathophysiology, and primary clinical symptoms. According to the authors, the information within the database was primarily sourced from scientific publications available in public databases like the National Center for Biotechnology Information (NCBI), ResearchGate, ScienceDirect, Wiley Online Library, Uniprot, and GenomeBrowser. GeneReviews, known for its expert contributions and rigorous review process, served as a primary resource for general polyQ disease information. The database also references the book “Polyglutamine Disorders” by C. Nobrega and L. Pereira de Almeida [334], which consolidates recent findings and expert knowledge in the field. Additionally, the database includes sections on clinical symptoms and neuropathological findings for each disease, and updates related to active clinical trials.

## 7. Concluding Remarks

Transient structural events and dynamic processes are hard to probe experimentally, hence computational studies play a pivotal role, offering a complementary perspective to experimental approaches. These studies, typically involving algorithms, QM and MD simulations, delve into the intricate molecular interactions and structural changes associated with polyQ expansions. They enable researchers to predict and visualize the complex folding patterns and aggregation propensities of proteins, which are challenging to capture through experimental means alone.

The behavior of protein (mis)folding and its tendency to aggregate are influenced by a range of factors. For instance, the isoelectric point marks a critical pH level where a protein’s net charge is neutral, impacting its solubility due to reduced repulsive charges. Elements like ionic strength and temperature also play vital roles, affecting protein charge interactions and stability. In this context, elevated temperatures can destabilize H-bonds and hydrophobic interactions, leading to protein unfolding and subsequent interactions. Moreover, an increased concentration of polyQ proteins can heighten the prevalence of aggregating interactions. Notably, changes in protein secondary structure, such as transitions from α-helices to β-sheets, are frequently observed precursors of polyQ aggregation.

The aggregation pattern—at atomic and molecular level—is based on the nucleation and growth model, where proteins initially form oligomeric aggregates that are transient and structurally diverse. These aggregates subsequently reorganize into more structured fibrils with a cross-β structure. However, the early aggregate stages of this process are still difficult to be characterized experimentally due to their short-lived and varied nature. Interestingly, the aggregation behavior of polyQ peptides is not always, and directly, influenced by factors like hydrophobicity, charge, and β-sheet affinity. These findings suggest a complex array of additional forces at play. This complexity is further underscored by the fact that any disruption in polyQ’s structural architecture leads to destabilization, along with both gain- and loss-of-function phenotypes, emphasizing the intricate structure–function relationship in polyQ diseases.

Experimental studies are vital as they explore scenarios beyond the limited environments often assumed in theoretical models. Moreover, they delve into the influence of environmental factors, going beyond pH and temperature, on protein aggregation. Importantly, experimental research sheds light on the cellular consequences of polyQ expansions, revealing their impact on critical functions like transcription, autophagy, and synaptic transmission, thereby deepening our understanding of the polyQ-related disease mechanisms at a cellular level.

While some studies indicate that large polyQ aggregates might be toxic, others propose that specific monomeric shapes or soluble oligomers are the actual culprits behind cell death. Another theory suggests that the polyQ segments might disrupt the functions of other cell components, leading to cellular demise. Current research acknowledges the impact of the polyQ region on protein aggregation, yet emphasizes that this process varies based on the protein environment. Experiments have shown that the mutation that causes polyQ expansion alters the structure and function of the host proteins. This leads to uncontrolled, abnormal interactions with critical cellular components, disrupting various processes like transcription and proteasomal degradation. Animal model studies have also shown that the expanded polyQ stretch itself causes neuron degeneration and motor disturbances. Furthermore, various cellular proteins, including chaperones and transcription factors, are found within inclusion bodies, suggesting that their sequestration might also contribute to neuronal dysfunction and cell loss.

Thus, while structural data on polyQ-rich peptides and proteins provide valuable insights, the unique properties and functions of each protein implicated in polyQ diseases complicate the development of a ‘universal structural hypothesis’ explaining how various polyQ conformations contribute to neurotoxicity.

Finally, in the context of elucidating the emerging patterns of polyQ diseases, what should we consider first—the theory or the experiment? Clearly, in unraveling the complexities of polyQ disorders, the interplay between theory and experimental implementation is essential. Theoretically, a robust framework provides hypotheses and models regarding protein behavior and disease mechanisms. These theories guide the experimental design, which in turn validates or refines theoretical models. Conversely, experimental findings can inspire new theoretical concepts and integrate adjustments into the existing ones. Therefore, neither theory nor experiment holds absolute precedence; rather, their integration forms a reciprocal and dynamic process essential to advancing our understanding on polyQ pathology. This synergistic approach—where theory and experiment inform and enhance each other—is crucial for developing effective therapeutic strategies in order to tackle these enigmatic disorders.

## Figures and Tables

**Table 2:** Normal and pathogenic CAG repeat ranges reported in 2013 [116], 2017 [85], 2020 [115], 2021 [117] and 2022 [125] in the context of polyQ-related disorders. The intermediate/pre-mutation repeat ranges were not considered.

PolyQ Disease	Normal CAG Repeats					Pathogenic CAG Repeats				
	Reports from 2013	Reports from 2017	Reports from 2020	Reports from 2021	Reports from 2022	Reports from 2013	Reports from 2017	Reports from 2020	Reports from 2021	Reports from 2022
HD	7–35	6–35	6–35	-	10–26	36–170	36–180	36–250	-	36–250
SBMA	11–35	9–36	11–34	-	5–34	38–62	38–65	38–62	-	37–70
DRPLA	7–34	6–36	3–35	-	7–35	48–93	49–88	49–88	-	49–88
SCA1	27–36	6–39	6–44	6–35	6–35	39–82	39–83	39–83	41–89	39–91
SCA2	15–32	14–32	15–31	17–29	14–31	36–64	32–200	34–200	37–100 +	33–500
SCA3	12–43	12–41	12–41	7–44	11–44	52–86	55–84	55–84	55–89	60–87
SCA6	8–14	4–19	4–17	4–18	4–18	20–33	20–33	20–33	21–30	20–33
SCA7	7–35	4–35	4–35	7–19	4–19	38–200	37–306	37–306	36–400 +	34–460
SCA17	24–44	25–44	25–42	25–42	25–41	45–63	46–63	45–63	47–66	46–55

The symbol ‘+’ indicates ‘more than’, however the exact upper limit of CAG repeats is not specified.

**Table 3:** Autophagy processes in polyQ-related neurodegeneration (adapted from Cortes et al. [102]).

PolyQ Disease	Autophagic Dysfunctions
HD	AVs accumulation Inhibition of autophagy signaling Defective cargo recognition
SBMA	AVs accumulation Impaired autophagy flux TFEB dysregulation
DRPLA	Impaired lysosomal degradation
SCA1	Increased levels of LC3II Cytoplasmic vacuoles accumulation
SCA2	Altered levels of SQSTM1 and LC3B
SCA3	Impaired autophagy induction Autophagy flux defects
SCA6	Impaired autophagy induction Lysosomal proteins within aggregates
SCA7	AVs accumulation Impaired p53 signaling
SCA17	Decreased autophagy activation via HMGB1 sequestration

AVs—autophagic vesicles, TFEB—transcription factor EB, LC3II—microtubule-associated proteins 1A/1B light chain 3B form II, SQSTM1—gene that encodes sequestosome-1 protein, LC3B—microtubule-associated proteins 1A/1B light chain 3B, p53—tumor suppressor protein (transcription factor activated in response to diverse stresses), and HMGB1—high mobility group box 1 (a chromatin-associated protein that regulates autophagy in response to oxidative stress).

**Table 4:** Differences and similarities between polyQ-related disorders.

Differences					Similarities
Features	SCAs	HD	SBMA	DRPLA	PolyQ-Disorders
Inheritance	Autosomal dominant	Autosomal dominant	X-linked recessive	Autosomal dominant	CAG expansion [127,128,129,130,131,132,133,134,135,136,137,154,155,156,157,184,185,186,187,188,205,206,207,208,209,210,211,212,213]
Disease pathology	Mitochondrial dysfunction, protein aggregation [130,135,136,137]	Protein aggregation, mitochondrial disruption [154,155,156,157]	Protein misfolding, transcriptional interference [184,185,186,187,188]	Protein accumulation, impaired lysosomal function [205,206,207,208,209,210,211,212,213]	Host proteins aggregations [135,136,137,154,155,156,157,184,185,186,187,188,205,206,207,208,209,210,211,212,213]
Genetic mechanism	CAG repeat expansions, aggregation propensity [130,135,136,137]	Expanded CAG repeats in HTT gene, toxic GOF/LOF [154,155,156,157]	PolyQ expansions in AR gene, GOF/LOF mechanisms [184,185,186,187,188]	Expanded CAG repeats in ATN1 gene, GOF mechanism [205,206,207,208,209,210,211,212,213]	Impaired autophagy and mitochondrial dysfunction [139,140,141,142,143,175,176,177,191,198,199,213]
Proteotoxicity	Increased nuclear import, faulty clearance by UPS [147,148,149,150,151]	Accumulation in nucleus, toxic oligomers [115,160,161]	Misfolded proteins in nucleus, proteotoxic species [190,191]	Diffuse accumulation in neuronal nuclei [212]	GOF and LOF toxicity [127,128,129,130,131,132,133,134,135,136,137,138,139,140,141,142,143,144,145,146,147,148,159,160,161,185,186,187,188,205,206,207,208,209,210,211,212,213]
Affected CNS regions	Cerebellum, brainstem, spinal cord, basal ganglia, peripheral and autonomic nerves [128,129,130,131]	Striatum, cerebral cortex [170,171]	Lower motor neurons, skeletal muscle [186,187]	Dentatorubral and pallidoluysian systems, cerebral white matter [205,206,207,208,209,210,211]	Aberrant PPIs [147,148,149,150,151,154,155,156,157,184,185,186,187,188,205,206,207,208,209,210,211,212,213]

SCAs—spinocerebellar ataxias; HD—Huntington’s disease; SBMA—spinal and bulbar muscular atrophy; DRPLA—dentatorubral pallidoluysian atrophy; polyQ—polyglutamine; CAG—cytosine, adenine, guanine; HTT—huntingtin gene; AR—Androgen receptor; ATN1—Atrophin-1; GOF—gain of function; LOF—loss-of-function; PPIs—protein–protein interactions; CNS—central nervous system.

## Abbreviations

polyQ	Polyglutamine
3D	Three-dimensional
H-bonds	Hydrogen bonds
2D	Two-dimensional
Cryo-EM	Cryogenic electron microscopy
NMR	Nuclear magnetic resonance
WT	Wild type
PSN	Protein structural network
P2P	Protein structural classification
PCN	Protein contact network
NAPS	Network analysis of protein structure
PPI	Protein–protein interaction
NNA	Neuronal network algorithm
PCA	Principal component analysis
vdW	Van der Waals
DNM	Dynamics network models
ENM	Elastic network models
PEN	Protein energy networks
PRS	Perturbation response scanning
AAN	Amino acid network
SAXS	Small-angle X-ray scattering
MS	Mass spectrometry
AFM	Atomic force microscopy
smFRET	Single-molecule fluorescence resonance energy transfer
ML	Machine learning
AI	Artificial intelligence
FFs	Force fields
Q	Glutamine
AR	Androgen receptor
SBMA	Spinal and bulbar muscular atrophy
SCA	Spinocerebellar ataxia
HD	Huntington’s disease
DRPLA	Dentatorubral pallidoluysian atrophy
CAG	Cytosine adenine guanine
CAA	Cytosine adenine adenine
CCG	Cytosine cytosine guanine
CCA	Cytosine cytosine adenine
CAT	Cytosine adenine thymine
CAC	Cytosine adenine cytosine
CTA	Cytosine thymine adenine
CTG	Cytosine thymine guanine
CNS	Central nervous system
ATP	Adenosine 5′-triphosphate
GOF	Gain of function
LOF	Loss of function
SSM	Slipped strand mispairing
RNA	Ribonucleic acid
TF	Transcription factor
AV	Autophagic vesicle
UPS	Ubiquitin–proteasome system
HTT	Huntingtin
mHTT	Mutant huntingtin
wtHTT	Wild-type huntingtin
IT15	Interesting transcript 15
PRD	Proline-rich domain
MSNs	Medium spiny neurons
ATPase	Adenosine triphosphatase
NTD	Amino-terminal transactivation domain
DBD	DNA-binding domain
LBD	Ligand-binding domain
LC3	Microtubule-associated protein 1A/1B-light chain 3
TFEB	Transcription factor EB
ATN1	Atrophin-1
MD	Molecular dynamics
REMD	Replica exchange MD
CG	Coarse grain
MC	Monte Carlo simulation
PPII	Poly-l-proline type II
QM	Quantum mechanics
MM	Molecular mechanics
polyP	Polyproline
polyA	Polyalanine
FLIM	Fluorescence lifetime imaging
FLIM-FRET	FLIM microscopy detection of Förster resonance energy transfer
CD	Circular dichroism
DLS	Dynamic light scattering
FTIR	Fourier transform infrared
NPCs	Neural progenitor cells
iPSCs	Induced pluripotent stem cells
MSCs	Mesenchymal stem cells
ASOs	Antisense oligonucleotides
CSF	Cerebrospinal fluid
AAV	Adeno-associated virus
siRNA	Small interfering RNA
ICV	Intracerebroventricular
CaM	Calmodulin
UVRR	Ultra-Violet Resonance Raman

## References

[1] Huntley M.A., Golding G.B. (2002). Simple sequences are rare in the Protein Data Bank. Proteins.

[2] Karlin S., Burge C. (1996). Trinucleotide repeats and long homopeptides in genes and proteins associated with nervous system disease and development. Proc. Natl. Acad. Sci. USA.

[3] Jorda J., Kajava A.V. (2010). Protein Homorepeats. Advances in Protein Chemistry and Structural Biology.

[4] Fondon J.W., Garner H.R. (2004). Molecular origins of rapid and continuous morphological evolution. Proc. Natl. Acad. Sci. USA.

[5] Brouwer J.R., Willemsen R., Oostra B.A. (2009). Microsatellite repeat instability and neurological disease. Bioessays.

[6] Elena-Real C.A., Mier P., Sibille N., Andrade-Navarro M.A., Bernadó P. (2023). Structure–function relationships in protein homorepeats. Curr. Opin. Struct. Biol..

[7] Chavali S., Singh A.K., Santhanam B., Babu M.M. (2020). Amino acid homorepeats in proteins. Nat. Rev. Chem..

[8] Pereira D., Cunha-Santos J., Vasconcelos-Ferreira A., Duarte-Neves J., Onofre I., Carmona V., Aveleira C.A., Lopes S.M., Lobo D.D., Martins I.M. (2023). Nuclear Aging in Polyglutamine-Induced Neurodegeneration. bioRxiv.

[9] Bhagavan N.V., Ha C.-E. (2015). Three-Dimensional Structure of Proteins and Disorders of Protein Misfolding. Essentials of Medical Biochemistry.

[10] Chirigati F. (2021). Predicting protein structure from cryo-EM data. Nat. Comput. Sci..

[11] Prabantu V.M., Gadiyaram V., Vishveshwara S., Srinivasan N. (2022). Understanding structural variability in proteins using protein structural networks. Curr. Res. Struct. Biol..

[12] Burley S.K., Berman H.M., Duarte J.M., Feng Z., Flatt J.W., Hudson B.P., Lowe R., Peisach E., Piehl D.W., Rose Y. (2022). Protein Data Bank: A Comprehensive Review of 3D Structure Holdings and Worldwide Utilization by Researchers, Educators, and Students. Biomolecules.

[13] Shoemaker S.C., Ando N. (2018). X-rays in the Cryo-Electron Microscopy Era: Structural Biology’s Dynamic Future. Biochemistry.

[14] Gauto D.F., Estrozi L.F., Schwieters C.D., Effantin G., Macek P., Sounier R., Sivertsen A.C., Schmidt E., Kerfah R., Mas G. (2019). Integrated NMR and cryo-EM atomic-resolution structure determination of a half-megadalton enzyme complex. Nat. Commun..

[15] Ramachandran G.N., Ramakrishnan C., Sasisekharan V. (1963). Stereochemistry of polypeptide chain configurations. J. Mol. Biol..

[16] Yang G., Hong N., Baier F., Jackson C.J., Tokuriki N. (2016). Conformational Tinkering Drives Evolution of a Promiscuous Activity through Indirect Mutational Effects. Biochemistry.

[17] Rajasekaran N., Suresh S., Gopi S., Raman K., Naganathan A.N. (2017). A General Mechanism for the Propagation of Mutational Effects in Proteins. Biochemistry.

[18] Guarnera E., Berezovsky I.N. (2019). On the perturbation nature of allostery: Sites, mutations, and signal modulation. Curr. Opin. Struct. Biol..

[19] Guarnera E., Berezovsky I.N. (2019). Toward Comprehensive Allosteric Control over Protein Activity. Structure.

[20] Guarnera E., Berezovsky I.N. (2020). Allosteric drugs and mutations: Chances, challenges, and necessity. Curr. Opin. Struct. Biol..

[21] Naganathan A.N. (2019). Modulation of allosteric coupling by mutations: From protein dynamics and packing to altered native ensembles and function. Curr. Opin. Struct. Biol..

[22] Prabantu V.M., Naveenkumar N., Srinivasan N. (2021). Influence of Disease-Causing Mutations on Protein Structural Networks. Front. Mol. Biosci..

[23] Rose G.D. (2019). Ramachandran maps for side chains in globular proteins. Proteins.

[24] Gromiha M.M., Selvaraj S. (2004). Inter-residue interactions in protein folding and stability. Prog. Biophys. Mol. Biol..

[25] Baker D. (2000). A surprising simplicity to protein folding. Nature.

[26] Bhattacharya S., Roche R., Shuvo M.H., Bhattacharya D. (2021). Recent Advances in Protein Homology Detection Propelled by Inter-Residue Interaction Map Threading. Front. Mol. Biosci..

[27] Yang J., Anishchenko I., Park H., Peng Z., Ovchinnikov S., Baker D. (2020). Improved protein structure prediction using predicted interresidue orientations. Proc. Natl. Acad. Sci. USA.

[28] Vijayabaskar M.S., Vishveshwara S. (2010). Interaction Energy Based Protein Structure Networks. Biophys. J..

[29] Taylor N.R. (2013). Small world network strategies for studying protein structures and binding. Comput. Struct. Biotechnol. J..

[30] Bhattacharyya M., Ghosh S., Vishveshwara S. (2015). Protein Structure and Function: Looking through the Network of Side-Chain Interactions. Curr. Protein Pept. Sci..

[31] Costanzi S. (2015). Topological Analyses of Protein-Ligand Binding: A Network Approach. Curr. Protein Pept. Sci..

[32] Guarnera E., Tan Z.W., Zheng Z., Berezovsky I.N. (2017). AlloSigMA: Allosteric signaling and mutation analysis server. Bioinformatics.

[33] Newaz K., Ghalehnovi M., Rahnama A., Antsaklis P.J., Milenković T. (2020). Network-based protein structural classification. R. Soc. Open Sci..

[34] Grewal R., Roy S. (2015). Modeling proteins as residue interaction networks. Protein Pept. Lett..

[35] Yan W., Zhou J., Sun M., Chen J., Hu G., Shen B. (2014). The construction of an amino acid network for understanding protein structure and function. Amino Acids.

[36] Di Paola L., De Ruvo M., Paci P., Santoni D., Giuliani A. (2013). Protein Contact Networks: An Emerging Paradigm in Chemistry. Chem. Rev..

[37] Greene L.H. (2012). Protein structure networks. Brief. Funct. Genom..

[38] Brinda K.V., Vishveshwara S. (2005). A Network Representation of Protein Structures: Implications for Protein Stability. Biophys. J..

[39] Böde C., Kovács I.A., Szalay M.S., Palotai R., Korcsmáros T., Csermely P. (2007). Network analysis of protein dynamics. FEBS Lett..

[40] Amitai G., Shemesh A., Sitbon E., Shklar M., Netanely D., Venger I., Pietrokovski S. (2004). Network Analysis of Protein Structures Identifies Functional Residues. J. Mol. Biol..

[41] Tse A., Verkhivker G.M. (2015). Molecular Dynamics Simulations and Structural Network Analysis of c-Abl and c-Src Kinase Core Proteins: Capturing Allosteric Mechanisms and Communication Pathways from Residue Centrality. J. Chem. Inf. Model..

[42] Sistla R.K., Brinda K.V., Vishveshwara S. (2005). Identification of domains and domain interface residues in multidomain proteins from graph spectral method. Proteins.

[43] Chakrabarty B., Parekh N. Analysis of graph centrality measures for identifying Ankyrin repeats. Proceedings of the 2012 World Congress on Information and Communication Technologies.

[44] Chakrabarty B., Parekh N. (2016). NAPS: Network Analysis of Protein Structures. Nucleic Acids Res..

[45] Bastolla U., Dehouck Y., Echave J. (2017). What evolution tells us about protein physics, and protein physics tells us about evolution. Curr. Opin. Struct. Biol..

[46] Dunker A.K., Silman I., Uversky V.N., Sussman J.L. (2008). Function and structure of inherently disordered proteins. Curr. Opin. Struct. Biol..

[47] Fuxreiter M. (2018). Towards a Stochastic Paradigm: From Fuzzy Ensembles to Cellular Functions. Molecules.

[48] Copley S.D. (2017). Shining a light on enzyme promiscuity. Curr. Opin. Struct. Biol..

[49] Manglik A., Kim T.H., Masureel M., Altenbach C., Yang Z., Hilger D., Lerch M.T., Kobilka T.S., Thian F.S., Hubbell W.L. (2015). Structural Insights into the Dynamic Process of β 2 -Adrenergic Receptor Signaling. Cell.

[50] Ross E.D., Baxa U., Wickner R.B. (2004). Scrambled Prion Domains Form Prions and Amyloid. Mol. Cell. Biol..

[51] Franzmann T.M., Jahnel M., Pozniakovsky A., Mahamid J., Holehouse A.S., Nüske E., Richter D., Baumeister W., Grill S.W., Pappu R.V. (2018). Phase separation of a yeast prion protein promotes cellular fitness. Science.

[52] Pacini L., Dorantes-Gilardi R., Vuillon L., Lesieur C. (2021). Mapping Function from Dynamics: Future Challenges for Network-Based Models of Protein Structures. Front. Mol. Biosci..

[53] Jaffe E.K. (2020). Wrangling Shape-Shifting Morpheeins to Tackle Disease and Approach Drug Discovery. Front. Mol. Biosci..

[54] Parisi G., Zea D.J., Monzon A.M., Marino-Buslje C. (2015). Conformational diversity and the emergence of sequence signatures during evolution. Curr. Opin. Struct. Biol..

[55] Surpeta B., Sequeiros-Borja C., Brezovsky J. (2020). Dynamics, a Powerful Component of Current and Future in Silico Approaches for Protein Design and Engineering. Int. J. Mol. Sci..

[56] Leitner D.M., Yamato T., Parrill A.L., Lipkowitz K.B. (2018). Mapping Energy Transport Networks in Proteins. Reviews in Computational Chemistry.

[57] Liang Z., Hu J., Yan W., Jiang H., Hu G., Luo C. (2018). Deciphering the role of dimer interface in intrinsic dynamics and allosteric pathways underlying the functional transformation of DNMT3A. Biochim. Biophys. Acta (BBA)-Gen. Subj..

[58] Ponzoni L., Bahar I. (2018). Structural dynamics is a determinant of the functional significance of missense variants. Proc. Natl. Acad. Sci. USA.

[59] Bourgeat L., Pacini L., Serghei A., Lesieur C. (2021). Experimental diagnostic of sequence-variant dynamic perturbations revealed by broadband dielectric spectroscopy. Structure.

[60] Gheeraert A., Pacini L., Batista V.S., Vuillon L., Lesieur C., Rivalta I. (2019). Exploring Allosteric Pathways of a V-Type Enzyme with Dynamical Perturbation Networks. J. Phys. Chem. B.

[61] Melo M.C.R., Bernardi R.C., de la Fuente-Nunez C., Luthey-Schulten Z. (2020). Generalized correlation-based dynamical network analysis: A new high-performance approach for identifying allosteric communications in molecular dynamics trajectories. J. Chem. Phys..

[62] Liang Z., Verkhivker G.M., Hu G. (2020). Integration of network models and evolutionary analysis into high-throughput modeling of protein dynamics and allosteric regulation: Theory, tools and applications. Brief. Bioinform..

[63] Di Paola L., Leitner D.M. (2021). Network models of biological adaptation at the molecular scale. Phys. Life Rev..

[64] Wingert B., Krieger J., Li H., Bahar I. (2021). Adaptability and specificity: How do proteins balance opposing needs to achieve function?. Curr. Opin. Struct. Biol..

[65] Dorantes-Gilardi R., Bourgeat L., Pacini L., Vuillon L., Lesieur C. (2018). In proteins, the structural responses of a position to mutation rely on the Goldilocks principle: Not too many links, not too few. Phys. Chem. Chem. Phys..

[66] Salamanca Viloria J., Allega M.F., Lambrughi M., Papaleo E. (2017). An optimal distance cutoff for contact-based Protein Structure Networks using side-chain centers of mass. Sci. Rep..

[67] Muñoz V., Cerminara M. (2016). When fast is better: Protein folding fundamentals and mechanisms from ultrafast approaches. Biochem. J..

[68] Gupta A., Singh A., Ahmad N., Singh T.P., Sharma S., Sharma P. (2022). Experimental techniques to study protein dynamics and conformations. Advances in Protein Molecular and Structural Biology Methods.

[69] Noé F., De Fabritiis G., Clementi C. (2020). Machine learning for protein folding and dynamics. Curr. Opin. Struct. Biol..

[70] Robustelli P., Piana S., Shaw D.E. (2018). Developing a molecular dynamics force field for both folded and disordered protein states. Proc. Natl. Acad. Sci. USA.

[71] Davtyan A., Schafer N.P., Zheng W., Clementi C., Wolynes P.G., Papoian G.A. (2012). AWSEM-MD: Protein Structure Prediction Using Coarse-Grained Physical Potentials and Bioinformatically Based Local Structure Biasing. J. Phys. Chem. B.

[72] Kluber A., Burt T.A., Clementi C. (2018). Size and topology modulate the effects of frustration in protein folding. Proc. Natl. Acad. Sci. USA.

[73] Chavali S., Chavali P.L., Chalancon G., de Groot N.S., Gemayel R., Latysheva N.S., Ing-Simmons E., Verstrepen K.J., Balaji S., Babu M.M. (2017). Constraints and consequences of the emergence of amino acid repeats in eukaryotic proteins. Nat. Struct. Mol. Biol..

[74] Lobanov M.Y., Galzitskaya O.V. (2012). Occurrence of disordered patterns and homorepeats in eukaryotic and bacterial proteomes. Mol. BioSyst..

[75] Faber P.W., Kuiper G., van Rooij H., van der Korput J., Brinkmann A., Trapman J. (1989). The N-terminal domain of the human androgen receptor is encoded by one, large exon. Mol. Cell. Endocrinol..

[76] La Spada A.R., Roling D.B., Harding A.E., Warner C.L., Spiegel R., Hausmanowa-Petrusewicz I., Yee W.-C., Fischbeck K.H. (1992). Meiotic stability and genotype–phenotype correlation of the trinucleotide repeat in X–linked spinal and bulbar muscular atrophy. Nat. Genet..

[77] Gatchel J.R., Zoghbi H.Y. (2005). Diseases of Unstable Repeat Expansion: Mechanisms and Common Principles. Nat. Rev. Genet..

[78] Almeida B., Fernandes S., Abreu I.A., Macedo-Ribeiro S. (2013). Trinucleotide Repeats: A Structural Perspective. Front. Neurol..

[79] Mier P., Andrade-Navarro M.A. (2021). Between Interactions and Aggregates: The PolyQ Balance. Genome Biol. Evol..

[80] Minakawa E.N., Nagai Y. (2021). Protein Aggregation Inhibitors as Disease-Modifying Therapies for Polyglutamine Diseases. Front. Neurosci..

[81] Lieberman A.P., Shakkottai V.G., Albin R.L. (2019). Polyglutamine Repeats in Neurodegenerative Diseases. Annu. Rev. Pathol. Mech. Dis..

[82] Hirunagi T., Sahashi K., Meilleur K.G., Katsuno M. (2022). Nucleic Acid-Based Therapeutic Approach for Spinal and Bulbar Muscular Atrophy and Related Neurological Disorders. Genes.

[83] Sahashi K., Katsuno M. (2018). Pathogenesis of Polyglutamine Diseases. Encyclopedia of Life Sciences.

[84] Rudich P., Watkins S., Lamitina T. (2020). PolyQ-independent toxicity associated with novel translational products from CAG repeat expansions. PLoS ONE.

[85] Takeuchi T., Nagai Y. (2017). Protein Misfolding and Aggregation as a Therapeutic Target for Polyglutamine Diseases. Brain Sci..

[86] Nagai Y., Minakawa E.N., Wada K. (2015). Drug Development for Neurodegenerative Diseases. Neurodegenerative Disorders as Systemic Diseases.

[87] Paulson H. (2018). Repeat expansion diseases. Handbook of Clinical Neurology.

[88] Stoyas C.A., La Spada A.R. (2018). The CAG–polyglutamine repeat diseases: A clinical, molecular, genetic, and pathophysiologic nosology. Handbook of Clinical Neurology.

[89] Totzeck F., Andrade-Navarro M.A., Mier P. (2017). The Protein Structure Context of PolyQ Regions. PLoS ONE.

[90] Mier P., Elena-Real C., Urbanek A., Bernadó P., Andrade-Navarro M.A. (2020). The importance of definitions in the study of polyQ regions: A tale of thresholds, impurities and sequence context. Comput. Struct. Biotechnol. J..

[91] Ramazzotti M., Monsellier E., Kamoun C., Degl’Innocenti D., Melki R. (2012). Polyglutamine repeats are associated to specific sequence biases that are conserved among eukaryotes. PLoS ONE.

[92] Shao J., Diamond M.I. (2007). Polyglutamine diseases: Emerging concepts in pathogenesis and therapy. Hum. Mol. Genet..

[93] Katti M.V., Ranjekar P.K., Gupta V.S. (2001). Differential Distribution of Simple Sequence Repeats in Eukaryotic Genome Sequences. Mol. Biol. Evol..

[94] Karlin S., Brocchieri L., Bergman A., Mrázek J., Gentles A.J. (2002). Amino acid runs in eukaryotic proteomes and disease associations. Proc. Natl. Acad. Sci. USA.

[95] Garden G.A., Libby R.T., Fu Y.-H., Kinoshita Y., Huang J., Possin D.E., Smith A.C., Martinez R.A., Fine G.C., Grote S.K. (2002). Polyglutamine-Expanded Ataxin-7 Promotes Non-Cell-Autonomous Purkinje Cell Degeneration and Displays Proteolytic Cleavage in Ataxic Transgenic Mice. J. Neurosci..

[96] Kubodera T., Yokota T., Ohwada K., Ishikawa K., Miura H., Matsuoka T., Mizusawa H. (2003). Proteolytic cleavage and cellular toxicity of the human α1A calcium channel in spinocerebellar ataxia type 6. Neurosci. Lett..

[97] Friedman M.J., Wang C.-E., Li X.-J., Li S. (2008). Polyglutamine Expansion Reduces the Association of TATA-binding Protein with DNA and Induces DNA Binding-independent Neurotoxicity. J. Biol. Chem..

[98] Jimenez-Sanchez M., Thomson F., Zavodszky E., Rubinsztein D.C. (2012). Autophagy and polyglutamine diseases. Prog. Neurobiol..

[99] Ren H., Hao Z., Wang G., Le W. (2020). Autophagy and Polyglutamine Disease. Autophagy: Biology and Diseases.

[100] Boland B., Kumar A., Lee S., Platt F.M., Wegiel J., Yu W.H., Nixon R.A. (2008). Autophagy induction and autophagosome clearance in neurons: Relationship to autophagic pathology in Alzheimer’s disease. J. Neurosci..

[101] La Spada A.R., Taylor J.P. (2010). Repeat expansion disease: Progress and puzzles in disease pathogenesis. Nat. Rev. Genet..

[102] Cortes C.J., La Spada A.R. (2015). Autophagy in polyglutamine disease: Imposing order on disorder or contributing to the chaos?. Mol. Cell. Neurosci..

[103] Son J.H., Shim J.H., Kim K.-H., Ha J.-Y., Han J.Y. (2012). Neuronal autophagy and neurodegenerative diseases. Exp. Mol. Med..

[104] Gu M., Gash M.T., Mann V.M., Javoy-Agid F., Cooper J.M., Schapira A.H.V. (1996). Mitochondrial defect in Huntington’s disease caudate nucleus. Ann. Neurol..

[105] Sorolla M.A., Reverter-Branchat G., Tamarit J., Ferrer I., Ros J., Cabiscol E. (2008). Proteomic and oxidative stress analysis in human brain samples of Huntington disease. Free Radic. Biol. Med..

[106] Stack E.C., Matson W.R., Ferrante R.J. (2008). Evidence of Oxidant Damage in Huntington’s Disease: Translational Strategies Using Antioxidants. Ann. N. Y. Acad. Sci..

[107] Jackson W.S. (2014). Selective vulnerability to neurodegenerative disease: The curious case of Prion Protein. Dis. Models Mech..

[108] Monaco A., Fraldi A. (2020). Protein Aggregation and Dysfunction of Autophagy-Lysosomal Pathway: A Vicious Cycle in Lysosomal Storage Diseases. Front. Mol. Neurosci..

[109] Ciechanover A., Kwon Y.T. (2015). Degradation of misfolded proteins in neurodegenerative diseases: Therapeutic targets and strategies. Exp. Mol. Med..

[110] Gallardo R., Ramakers M., De Smet F., Claes F., Khodaparast L., Khodaparast L., Couceiro J.R., Langenberg T., Siemons M., Nyström S. (2016). De novo design of a biologically active amyloid. Science.

[111] Watt N.T., Taylor D.R., Kerrigan T.L., Griffiths H.H., Rushworth J.V., Whitehouse I.J., Hooper N.M. (2012). Prion protein facilitates uptake of zinc into neuronal cells. Nat. Commun..

[112] You H., Tsutsui S., Hameed S., Kannanayakal T.J., Chen L., Xia P., Engbers J.D., Lipton S.A., Stys P.K., Zamponi G.W. (2012). Aβ neurotoxicity depends on interactions between copper ions, prion protein, and N-methyl-D-aspartate receptors. Proc. Natl. Acad. Sci. USA.

[113] Spevacek A.R., Evans E.G., Miller J.L., Meyer H.C., Pelton J.G., Millhauser G.L. (2013). Zinc drives a tertiary fold in the prion protein with familial disease mutation sites at the interface. Structure.

[114] Davies P., Watt K., Kelly S.M., Clark C., Price N.C., McEwan I.J. (2008). Consequences of poly-glutamine repeat length for the conformation and folding of the androgen receptor amino-terminal domain. J. Mol. Endocrinol..

[115] Tunalı E.N., Ersoy Tunalı N. (2021). Molecular Mechanisms of Polyglutamine Pathology and Lessons Learned from Huntington’s Disease. Neurodegenerative Diseases-Molecular Mechanisms and Current Therapeutic Approaches.

[116] Margulis B.A., Vigont V., Lazarev V.F., Kaznacheyeva E.V., Guzhova I.V. (2013). Pharmacological protein targets in polyglutamine diseases: Mutant polypeptides and their interactors. FEBS Lett..

[117] McIntosh C.S., Li D., Wilton S.D., Aung-Htut M.T. (2021). Polyglutamine Ataxias: Our Current Molecular Understanding and What the Future Holds for Antisense Therapies. Biomedicines.

[118] Levinson G., Gutman G.A. (1987). Slipped-strand mispairing: A major mechanism for DNA sequence evolution. Mol. Biol. Evol..

[119] Sweeney P., Park H., Baumann M., Dunlop J., Frydman J., Kopito R., McCampbell A., Leblanc G., Venkateswaran A., Nurmi A. (2017). Protein misfolding in neurodegenerative diseases: Implications and strategies. Transl. Neurodegener..

[120] Zoghbi H.Y., Orr H.T. (1999). Polyglutamine diseases: Protein cleavage and aggregation. Curr. Opin. Neurobiol..

[121] Kuiper E.F.E., De Mattos E.P., Jardim L.B., Kampinga H.H., Bergink S. (2017). Chaperones in Polyglutamine Aggregation: Beyond the Q-Stretch. Front. Neurosci..

[122] Nath S.R., Lieberman A.P. (2017). The Ubiquitination, Disaggregation and Proteasomal Degradation Machineries in Polyglutamine Disease. Front. Mol. Neurosci..

[123] Cattaneo E. (2001). Loss of normal huntingtin function: New developments in Huntington’s disease research. Trends Neurosci..

[124] Saudou F., Finkbeiner S., Devys D., Greenberg M.E. (1998). Huntingtin Acts in the Nucleus to Induce Apoptosis but Death Does Not Correlate with the Formation of Intranuclear Inclusions. Cell.

[125] Karwacka M., Olejniczak M. (2022). Advances in Modeling Polyglutamine Diseases Using Genome Editing Tools. Cells.

[126] Naphade S., Tshilenge K.-T., Ellerby L.M. (2019). Modeling Polyglutamine Expansion Diseases with Induced Pluripotent Stem Cells. Neurotherapeutics.

[127] Orr H.T. (2012). Polyglutamine neurodegeneration: Expanded glutamines enhance native functions. Curr. Opin. Genet. Dev..

[128] Ashizawa T., Öz G., Paulson H.L. (2018). Spinocerebellar ataxias: Prospects and challenges for therapy development. Nat. Rev. Neurol..

[129] Almeida-Silva U.C., Hallak J.E.C., Júnior W.M., de Lima Osório F. (2013). Association between spinocerebellar ataxias caused by glutamine expansion and psychiatric and neuropsychological signals—A literature review. Am. J. Neurodegener. Dis..

[130] Klockgether T., Mariotti C., Paulson H.L. (2019). Spinocerebellar ataxia. Nat. Rev. Dis. Primers.

[131] Buijsen R.A.M., Toonen L.J., Gardiner S.L., van Roon-Mom W.M. (2019). Genetics, Mechanisms, and Therapeutic Progress in Polyglutamine Spinocerebellar Ataxias. Neurotherapeutics.

[132] Schöls L., Linnemann C., Globas C. (2008). Electrophysiology in spinocerebellar ataxias: Spread of disease and characteristic findings. Cerebellum.

[133] Liang L., Chen T., Wu Y. (2016). The electrophysiology of spinocerebellar ataxias. Neurophysiol. Clin..

[134] Ghanekar S.D., Kuo S.-H., Staffetti J.S., Zesiewicz T.A. (2022). Current and emerging treatment modalities for spinocerebellar ataxias. Expert Rev. Neurother..

[135] Paulson H.L., Shakkottai V.G., Clark H.B., Orr H.T. (2017). Polyglutamine spinocerebellar ataxias—From genes to potential treatments. Nat. Rev. Neurosci..

[136] Sullivan R., Yau W.Y., O’Connor E., Houlden H. (2019). Spinocerebellar ataxia: An update. J. Neurol..

[137] Bushart D.D., Shakkottai V.G. (2019). Ion channel dysfunction in cerebellar ataxia. Neurosci. Lett..

[138] Honti V., Vécsei L. (2005). Genetic and molecular aspects of spinocerebellar ataxias. Neuropsychiatr. Dis. Treat..

[139] Cornelius N., Wardman J.H., Hargreaves I.P., Neergheen V., Bie A.S., Tümer Z., Nielsen J.E., Nielsen T.T. (2017). Evidence of oxidative stress and mitochondrial dysfunction in spinocerebellar ataxia type 2 (SCA2) patient fibroblasts: Effect of coenzyme Q10 supplementation on these parameters. Mitochondrion.

[140] Hsu J.-Y., Jhang Y.-L., Cheng P.-H., Chang Y.-F., Mao S.-H., Yang H.-I., Lin C.-W., Chen C.-M., Yang S.-H. (2017). The Truncated C-terminal Fragment of Mutant ATXN3 Disrupts Mitochondria Dynamics in Spinocerebellar Ataxia Type 3 Models. Front. Mol. Neurosci..

[141] Kazachkova N., Raposo M., Montiel R., Cymbron T., Bettencourt C., Silva-Fernandes A., Duarte-Silva S., Maciel P., Lima M. (2013). Patterns of Mitochondrial DNA Damage in Blood and Brain Tissues of a Transgenic Mouse Model of Machado-Joseph Disease. Neurodegener. Dis..

[142] Ripolone M., Lucchini V., Ronchi D., Fagiolari G., Bordoni A., Fortunato F., Mondello S., Bonato S., Meregalli M., Torrente Y. (2018). Purkinje cell COX deficiency and mtDNA depletion in an animal model of spinocerebellar ataxia type 1. J. Neurosci. Res..

[143] Torres-Ramos Y., Montoya-Estrada A., Cisneros B., Tercero-Pérez K., León-Reyes G., Leyva-García N., Hernández-Hernández O., Magaña J.J. (2018). Oxidative Stress in Spinocerebellar Ataxia Type 7 Is Associated with Disease Severity. Cerebellum.

[144] Hara T., Nakamura K., Matsui M., Yamamoto A., Nakahara Y., Suzuki-Migishima R., Yokoyama M., Mishima K., Saito I., Okano H. (2006). Suppression of basal autophagy in neural cells causes neurodegenerative disease in mice. Nature.

[145] Komatsu M., Waguri S., Chiba T., Murata S., Iwata J.-I., Tanida I., Ueno T., Koike M., Uchiyama Y., Kominami E. (2006). Loss of autophagy in the central nervous system causes neurodegeneration in mice. Nature.

[146] Wong E., Cuervo A.M. (2010). Autophagy gone awry in neurodegenerative diseases. Nat. Neurosci..

[147] Lee D., Lee Y.-I., Lee Y.-S., Lee S.B. (2020). The Mechanisms of Nuclear Proteotoxicity in Polyglutamine Spinocerebellar Ataxias. Front. Neurosci..

[148] Chhangani D., Nukina N., Kurosawa M., Amanullah A., Joshi V., Upadhyay A., Mishra A. (2014). Mahogunin ring finger 1 suppresses misfolded polyglutamine aggregation and cytotoxicity. Biochim. Biophys. Acta (BBA)-Mol. Basis Dis..

[149] Chen Z.S., Wong AK Y., Cheng T.C., Koon A.C., Chan H.Y.E. (2019). FipoQ/ FBXO 33, a Cullin-1-based ubiquitin ligase complex component modulates ubiquitination and solubility of polyglutamine disease protein. J. Neurochem..

[150] Marinello M., Werner A., Giannone M., Tahiri K., Alves S., Tesson C., Dunnen W.D., Seeler J.-S., Brice A., Sittler A. (2018). SUMOylation by SUMO2 is implicated in the degradation of misfolded ataxin-7 via RNF4 in SCA7 models. Dis. Models Mech..

[151] Dhar N., Arsiwala A., Murali S., Kane R.S. (2020). “Trim”ming PolyQ proteins with engineered PML. Biotechnol. Bioeng..

[152] Ou Z., Luo M., Niu X., Chen Y., Xie Y., He W., Song B., Xian Y., Fan D., OuYang S. (2016). Autophagy Promoted the Degradation of Mutant ATXN3 in Neurally Differentiated Spinocerebellar Ataxia-3 Human Induced Pluripotent Stem Cells. BioMed Res. Int..

[153] Park S.-H., Kukushkin Y., Gupta R., Chen T., Konagai A., Hipp M.S., Hayer-Hartl M., Hartl F.U. (2013). PolyQ Proteins Interfere with Nuclear Degradation of Cytosolic Proteins by Sequestering the Sis1p Chaperone. Cell.

[154] Moldovean S.N., Chiş V. (2020). Molecular Dynamics Simulations Applied to Structural and Dynamical Transitions of the Huntingtin Protein: A Review. ACS Chem. Neurosci..

[155] Subramaniam S. (2019). Selective Neuronal Death in Neurodegenerative Diseases: The Ongoing Mystery. Yale J. Biol. Med..

[156] Perutz M.F., Johnson T., Suzuki M., Finch J.T. (1994). Glutamine repeats as polar zippers: Their possible role in inherited neurodegenerative diseases. Proc. Natl. Acad. Sci. USA.

[157] Kahlem P., Green H., Djian P. (1998). Transglutaminase Action Imitates Huntington’s Disease: Selective Polymerization of Huntingtin Containing Expanded Polyglutamine. Mol. Cell.

[158] Perutz M. (1994). Polar zippers: Their role in human disease. Protein Sci..

[159] Jarosińska O.D., Rüdiger S.G.D. (2021). Molecular Strategies to Target Protein Aggregation in Huntington’s Disease. Front. Mol. Biosci..

[160] Ambrose C.M., Duyao M.P., Barnes G., Bates G.P., Lin C.S., Srinidhi J., Baxendale S., Hummerich H., Lehrach H., Altherr M. (1994). Structure and expression of the Huntington’s disease gene: Evidence against simple inactivation due to an expanded CAG repeat. Somat. Cell Mol. Genet..

[161] White J.K., Auerbach W., Duyao M.P., Vonsattel J.-P., Gusella J.F., Joyner A.L., MacDonald M.E. (1997). Huntingtin is required for neurogenesis and is not impaired by the Huntington’s disease CAG expansion. Nat. Genet..

[162] Ashkenazi A., Bento C.F., Ricketts T., Vicinanza M., Siddiqi F., Pavel M., Squitieri F., Hardenberg M.C., Imarisio S., Menzies F.M. (2017). Polyglutamine tracts regulate beclin 1-dependent autophagy. Nature.

[163] Nollen E.A.A., Garcia S.M., van Haaften G., Kim S., Chavez A., Morimoto R.I., Plasterk R.H.A. (2004). Genome-wide RNA interference screen identifies previously undescribed regulators of polyglutamine aggregation. Proc. Natl. Acad. Sci. USA.

[164] Lee J.-M., Correia K., Loupe J., Kim K.-H., Barker D., Hong E.P., Chao M.J., Long J.D., Lucente D., Vonsattel J.P.G. (2019). CAG Repeat Not Polyglutamine Length Determines Timing of Huntington’s Disease Onset. Cell.

[165] Wright G.E.B., Collins J.A., Kay C., McDonald C., Dolzhenko E., Xia Q., Bečanović K., Drögemöller B.I., Semaka A., Nguyen C.M. (2019). Length of Uninterrupted CAG, Independent of Polyglutamine Size, Results in Increased Somatic Instability, Hastening Onset of Huntington Disease. Am. J. Hum. Genet..

[166] Martindale D., Hackam A., Wieczorek A., Ellerby L., Wellington C., McCutcheon K., Singaraja R., Kazemi-Esfarjani P., Devon R., Kim S.U. (1998). Length of huntingtin and its polyglutamine tract influences localization and frequency of intracellular aggregates. Nat. Genet..

[167] Chai Y., Koppenhafer S.L., Shoesmith S.J., Perez M.K., Paulson H.L. (1999). Evidence for Proteasome Involvement in Polyglutamine Disease: Localization to Nuclear Inclusions in SCA3/MJD and Suppression of Polyglutamine Aggregation in vitro. Hum. Mol. Genet..

[168] Peters M.F., Nucifora C.F., Kushia J., Seaman H.C., Cooper J.K., Herring W.J., Dawson V.L., Dawson T.M., Ross C.A. (1999). Nuclear Targeting of Mutant Huntingtin Increases Toxicity. Mol. Cell. Neurosci..

[169] Singh M.D., Chanu S.I., Sarkar S. (2016). Deciphering the Enigma of Human Poly(Q) Disorders: Contribution of Drosophila melanogaster: Singh MD et al. Contribution of Drosophila in human poly(Q) research. Int. J. Neurol. Res..

[170] Coppen E.M., Van Der Grond J., Roos R.A.C. (2018). Atrophy of the putamen at time of clinical motor onset in Huntington’s disease: A 6-year follow-up study. J. Clin. Mov. Disord..

[171] McColgan P., Seunarine K.K., Gregory S., Razi A., Papoutsi M., Long J.D., Mills J.A., Johnson E., Durr A., Roos R.A. (2017). Topological length of white matter connections predicts their rate of atrophy in premanifest Huntington’s disease. JCI Insight.

[172] Rikani A.A., Choudhry Z., Choudhry A.M., Rizvi N., Ikram H., Mobassarah N.J. (2014). The mechanism of degeneration of striatal neuronal subtypes in Huntington disease. Ann. Neurosci..

[173] Morigaki R., Goto S. (2017). Striatal Vulnerability in Huntington’s Disease: Neuroprotection Versus Neurotoxicity. Brain Sci..

[174] Kang R., Wang L., Sanders S.S., Zuo K., Hayden M.R., Raymond L.A. (2019). Altered Regulation of Striatal Neuronal N-Methyl-D-Aspartate Receptor Trafficking by Palmitoylation in Huntington Disease Mouse Model. Front. Synaptic Neurosci..

[175] Martinez-Vicente M., Talloczy Z., Wong E., Tang G., Koga H., Kaushik S., de Vries R., Arias E., Harris S., Sulzer D. (2010). Cargo recognition failure is responsible for inefficient autophagy in Huntington’s disease. Nat. Neurosci..

[176] Atwal R.S., Xia J., Pinchev D., Taylor J., Epand R.M., Truant R. (2007). Huntingtin has a membrane association signal that can modulate huntingtin aggregation, nuclear entry and toxicity. Hum. Mol. Genet..

[177] Kegel K.B., Kim M., Sapp E., McIntyre C., Castano J.G., Aronin N., DiFiglia M. (2000). Huntingtin Expression Stimulates Endosomal–Lysosomal Activity, Endosome Tubulation, and Autophagy. J. Neurosci..

[178] Dejager S., Bry-Gauillard H., Bruckert E., Eymard B., Salachas F., LeGuern E., Tardieu S., Chadarevian R., Giral P., Turpin G. (2002). A Comprehensive Endocrine Description of Kennedy’s Disease Revealing Androgen Insensitivity Linked to CAG Repeat Length. J. Clin. Endocrinol. Metab..

[179] Ravikumar B. (2002). Aggregate-prone proteins with polyglutamine and polyalanine expansions are degraded by autophagy. Hum. Mol. Genet..

[180] Ochaba J., Lukacsovich T., Csikos G., Zheng S., Margulis J., Salazar L., Mao K., Lau A.L., Yeung S.Y., Humbert S. (2014). Potential function for the Huntingtin protein as a scaffold for selective autophagy. Proc. Natl. Acad. Sci. USA.

[181] Rui Y.-N., Xu Z., Patel B., Cuervo A.M., Zhang S. (2015). HTT/Huntingtin in selective autophagy and Huntington disease: A foe or a friend within?. Autophagy.

[182] Rodriguez-Quiroga S.A., Gonzalez-Moron D., Garretto N., Kauffman M.A. (2013). Huntington’s disease masquerading as spinocerebellar ataxia. Case Rep..

[183] Franklin G.L., Camargo C.H.F., Meira A.T., Pavanelli G.M., Milano S.S., Germiniani F.B., Lima N.S.C., Raskin S., Barsottini O.G.P., Pedroso J.L. (2020). Is Ataxia an Underestimated Symptom of Huntington’s Disease?. Front. Neurol..

[184] Figiel M., Szlachcic W.J., Switonski P.M., Gabka A., Krzyzosiak W.J. (2012). Mouse Models of Polyglutamine Diseases: Review and Data Table. Part I. Mol. Neurobiol..

[185] Inagaki T., Hashizume A., Hijikata Y., Yamada S., Ito D., Kishimoto Y., Torii R., Sato H., Hirakawa A., Katsuno M. (2022). Development of a functional composite for the evaluation of spinal and bulbar muscular atrophy. Sci. Rep..

[186] Cortes C.J., La Spada A.R., Nóbrega C., Pereira De Almeida L. (2018). X-Linked Spinal and Bulbar Muscular Atrophy: From Clinical Genetic Features and Molecular Pathology to Mechanisms Underlying Disease Toxicity. Polyglutamine Disorders.

[187] Giorgetti E., Lieberman A.P. (2016). Polyglutamine androgen receptor-mediated neuromuscular disease. Cell. Mol. Life Sci..

[188] Kratter I.H., Finkbeiner S. (2010). PolyQ Disease: Too Many Qs, Too Much Function?. Neuron.

[189] Wyttenbach A. (2004). Role of Heat Shock Proteins During Polyglutamine Neurodegeneration: Mechanisms and Hypothesis. J. Mol. Neurosci..

[190] Jochum T., Ritz M.E., Schuster C., Funderburk S.F., Jehle K., Schmitz K., Brinkmann F., Hirtz M., Moss D., Cato A.C. (2012). Toxic and non-toxic aggregates from the SBMA and normal forms of androgen receptor have distinct oligomeric structures. Biochim. Biophys. Acta (BBA)-Mol. Basis Dis..

[191] Taylor J.P. (2003). Aggresomes protect cells by enhancing the degradation of toxic polyglutamine-containing protein. Hum. Mol. Genet..

[192] Kayed R., Head E., Thompson J.L., McIntire T.M., Milton S.C., Cotman C.W., Glabe C.G. (2003). Common Structure of Soluble Amyloid Oligomers Implies Common Mechanism of Pathogenesis. Science.

[193] Paramithiotis E., Pinard M., Lawton T., LaBoissiere S., Leathers V.L., Zou W.-Q., A Estey L., Lamontagne J., Lehto M.T., Kondejewski L.H. (2003). A prion protein epitope selective for the pathologically misfolded conformation. Nat. Med..

[194] Legleiter J., Lotz G.P., Miller J., Ko J., Ng C., Williams G.L., Finkbeiner S., Patterson P.H., Muchowski P.J. (2009). Monoclonal Antibodies Recognize Distinct Conformational Epitopes Formed by Polyglutamine in a Mutant Huntingtin Fragment. J. Biol. Chem..

[195] Miller J., Arrasate M., Brooks E., Libeu C.P., Legleiter J., Hatters D., Curtis J., Cheung K., Krishnan P., Mitra S. (2011). Identifying polyglutamine protein species in situ that best predict neurodegeneration. Nat. Chem. Biol..

[196] Klein F.A.C., Pastore A., Masino L., Zeder-Lutz G., Nierengarten H., Oulad-Abdelghani M., Altschuh D., Mandel J.-L., Trottier Y. (2007). Pathogenic and Non-pathogenic Polyglutamine Tracts Have Similar Structural Properties: Towards a Length-dependent Toxicity Gradient. J. Mol. Biol..

[197] Schaffar G., Breuer P., Boteva R., Behrends C., Tzvetkov N., Strippel N., Sakahira H., Siegers K., Hayer-Hartl M., Hartl F. (2004). Cellular Toxicity of Polyglutamine Expansion Proteins. Mol. Cell.

[198] Rusmini P., Bolzoni E., Crippa V., Onesto E., Sau D., Galbiati M., Piccolella M., Poletti A. (2010). Proteasomal and autophagic degradative activities in spinal and bulbar muscular atrophy. Neurobiol. Dis..

[199] Pandey U.B., Nie Z., Batlevi Y., McCray B.A., Ritson G.P., Nedelsky N.B., Schwartz S.L., DiProspero N.A., Knight M.A., Schuldiner O. (2007). HDAC6 rescues neurodegeneration and provides an essential link between autophagy and the UPS. Nature.

[200] Cortes C.J., Miranda H.C., Frankowski H., Batlevi Y., E Young J., Le A., Ivanov N., Sopher B.L., Carromeu C., Muotri A.R. (2014). Polyglutamine-expanded androgen receptor interferes with TFEB to elicit autophagy defects in SBMA. Nat. Neurosci..

[201] Doi H., Adachi H., Katsuno M., Minamiyama M., Matsumoto S., Kondo N., Miyazaki Y., Iida M., Tohnai G., Qiang Q. (2013). p62/SQSTM1 differentially removes the toxic mutant androgen receptor via autophagy and inclusion formation in a spinal and bulbar muscular atrophy mouse model. J. Neurosci..

[202] Montie H.L., Cho M.S., Holder L., Liu Y., Tsvetkov A.S., Finkbeiner S., Merry D.E. (2009). Cytoplasmic retention of polyglutamine-expanded androgen receptor ameliorates disease via autophagy in a mouse model of spinal and bulbar muscular atrophy. Hum. Mol. Genet..

[203] Sandri M., Coletto L., Grumati P., Bonaldo P. (2013). Misregulation of autophagy and protein degradation systems in myopathies and muscular dystrophies. J. Cell Sci..

[204] Yu Z., Wang A.M., Adachi H., Katsuno M., Sobue G., Yue Z., Robins D.M., Lieberman A.P. (2011). Macroautophagy is regulated by the UPR-mediator CHOP and accentuates the phenotype of SBMA mice. PLoS Genet..

[205] Chaudhry A., Anthanasiou-Fragkouli A., Houlden H. (2021). DRPLA: Understanding the natural history and developing biomarkers to accelerate therapeutic trials in a globally rare repeat expansion disorder. J. Neurol..

[206] Carroll L.S., Massey T.H., Wardle M., Peall K.J. (2018). Dentatorubral-pallidoluysian Atrophy: An Update. Tremor Other Hyperkinet. Mov..

[207] Hasegawa A., Ikeuchi T., Koike R., Matsubara N., Tsuchiya M., Nozaki H., Homma A., Idezuka J., Nishizawa M., Onodera O. (2010). Long-term disability and prognosis in dentatorubral-pallidoluysian atrophy: A correlation with CAG repeat length. Mov. Disord..

[208] Ikeuchi T., Koide R., Tanaka H., Onodera O., Igarashi S., Takahashi H., Kondo R., Ishikawa A., Tomoda A., Miike T. (1995). Dentatorubral-pallidoluysian atrophy: Clinical features are closely related to unstable expansions of trinucleotide (CAG) repeat. Ann. Neurol..

[209] Koide R., Ikeuchi T., Onodera O., Tanaka H., Igarashi S., Endo K., Takahashi H., Kondo R., Ishikawa A., Hayashi T. (1994). Unstable expansion of CAG repeat in hereditary dentatorubral–pallidoluysian atrophy (DRPLA). Nat. Genet..

[210] Maruyama S., Saito Y., Nakagawa E., Saito T., Komaki H., Sugai K., Sasaki M., Kumada S., Saito Y., Tanaka H. (2012). Importance of CAG repeat length in childhood-onset dentatorubral–pallidoluysian atrophy. J. Neurol..

[211] Sugiyama A., Sato N., Nakata Y., Kimura Y., Enokizono M., Maekawa T., Kondo M., Takahashi Y., Kuwabara S., Matsuda H. (2018). Clinical and magnetic resonance imaging features of elderly onset dentatorubral–pallidoluysian atrophy. J. Neurol..

[212] Tsuji S. (2012). Dentatorubral–pallidoluysian atrophy. Handbook of Clinical Neurology.

[213] Nisoli I., Chauvin J.P., Napoletano F., Calamita P., Zanin V., Fanto M., Charroux B. (2010). Neurodegeneration by polyglutamine Atrophin is not rescued by induction of autophagy. Cell Death Differ..

[214] Crick S.L., Jayaraman M., Frieden C., Wetzel R., Pappu R.V. (2006). Fluorescence correlation spectroscopy shows that monomeric polyglutamine molecules form collapsed structures in aqueous solutions. Proc. Natl. Acad. Sci. USA.

[215] Bennett M.J., Huey-Tubman K.E., Herr A.B., West A.P., Ross S.A., Bjorkman P.J. (2002). A linear lattice model for polyglutamine in CAG-expansion diseases. Proc. Natl. Acad. Sci. USA.

[216] Takahashi T., Kikuchi S., Katada S., Nagai Y., Nishizawa M., Onodera O. (2008). Soluble polyglutamine oligomers formed prior to inclusion body formation are cytotoxic. Hum. Mol. Genet..

[217] Takahashi Y., Okamoto Y., Popiel H.A., Fujikake N., Toda T., Kinjo M., Nagai Y. (2007). Detection of Polyglutamine Protein Oligomers in Cells by Fluorescence Correlation Spectroscopy. J. Biol. Chem..

[218] Takahashi T., Katada S., Onodera O. (2010). Polyglutamine Diseases: Where does Toxicity Come from? What is Toxicity? Where are We Going?. J. Mol. Cell Biol..

[219] Olshina M.A., Angley L.M., Ramdzan Y.M., Tang J., Bailey M.F., Hill A.F., Hatters D.M. (2010). Tracking Mutant Huntingtin Aggregation Kinetics in Cells Reveals Three Major Populations That Include an Invariant Oligomer Pool. J. Biol. Chem..

[220] Legleiter J., Mitchell E., Lotz G.P., Sapp E., Ng C., DiFiglia M., Thompson L.M., Muchowski P.J. (2010). Mutant Huntingtin Fragments Form Oligomers in a Polyglutamine Length-dependent Manner in Vitro and in Vivo. J. Biol. Chem..

[221] Sathasivam K., Lane A., Legleiter J., Warley A., Woodman B., Finkbeiner S., Paganetti P., Muchowski P.J., Wilson S., Bates G.P. (2010). Identical oligomeric and fibrillar structures captured from the brains of R6/2 and knock-in mouse models of Huntington’s disease. Hum. Mol. Genet..

[222] Nucifora L.G., Burke K.A., Feng X., Arbez N., Zhu S., Miller J., Yang G., Ratovitski T., Delannoy M., Muchowski P.J. (2012). Identification of Novel Potentially Toxic Oligomers Formed in Vitro from Mammalian-derived Expanded huntingtin Exon-1 Protein. J. Biol. Chem..

[223] Kim Y.E., Hosp F., Frottin F., Ge H., Mann M., Hayer-Hartl M., Hartl F.U. (2016). Soluble Oligomers of PolyQ-Expanded Huntingtin Target a Multiplicity of Key Cellular Factors. Mol. Cell.

[224] Kim M. (2014). Pathogenic polyglutamine expansion length correlates with polarity of the flanking sequences. Mol. Neurodegener..

[225] Hosp F., Gutiérrez-Ángel S., Schaefer M.H., Cox J., Meissner F., Hipp M.S., Hartl F.-U., Klein R., Dudanova I., Mann M. (2017). Spatiotemporal Proteomic Profiling of Huntington’s Disease Inclusions Reveals Widespread Loss of Protein Function. Cell Rep..

[226] Kwon M.J., Han M.H., Bagley J.A., Hyeon D.Y., Ko B.S., Lee Y.M., Cha I.J., Kim S.Y., Kim D.Y., Kim H.M. (2018). Coiled-coil structure-dependent interactions between polyQ proteins and Foxo lead to dendrite pathology and behavioral defects. Proc. Natl. Acad. Sci. USA.

[227] Eftekharzadeh B., Piai A., Chiesa G., Mungianu D., García J., Pierattelli R., Felli I.C., Salvatella X. (2016). Sequence Context Influences the Structure and Aggregation Behavior of a PolyQ Tract. Biophys. J..

[228] Urbanek A., Popovic M., Morató A., Estaña A., Elena-Real C.A., Mier P., Fournet A., Allemand F., Delbecq S., Andrade-Navarro M.A. (2020). Flanking Regions Determine the Structure of the Poly-Glutamine in Huntingtin through Mechanisms Common among Glutamine-Rich Human Proteins. Structure.

[229] De Chiara C., Pastore A. (2014). Kaleidoscopic protein–protein interactions in the life and death of ataxin-1: New strategies against protein aggregation. Trends Neurosci..

[230] Saunders H.M., Bottomley S.P. (2009). Multi-domain misfolding: Understanding the aggregation pathway of polyglutamine proteins. Protein Eng. Des. Sel..

[231] Punihaole D., Jakubek R.S., Workman R.J., Asher S.A. (2018). Interaction Enthalpy of Side Chain and Backbone Amides in Polyglutamine Solution Monomers and Fibrils. J. Phys. Chem. Lett..

[232] Natalello A., Frana A.M., Relini A., Apicella A., Invernizzi G., Casari C., Gliozzi A., Doglia S.M., Tortora P., Regonesi M.E. (2011). A Major Role for Side-Chain Polyglutamine Hydrogen Bonding in Irreversible Ataxin-3 Aggregation. PLoS ONE.

[233] Yushchenko T., Deuerling E., Hauser K. (2018). Insights into the Aggregation Mechanism of PolyQ Proteins with Different Glutamine Repeat Lengths. Biophys. J..

[234] Buchanan L.E., Carr J.K., Fluitt A.M., Hoganson A.J., Moran S.D., de Pablo J.J., Skinner J.L., Zanni M.T. (2014). Structural motif of polyglutamine amyloid fibrils discerned with mixed-isotope infrared spectroscopy. Proc. Natl. Acad. Sci. USA.

[235] Siu H.-W., Heck B., Kovermann M., Hauser K. (2021). Template-assisted design of monomeric polyQ models to unravel the unique role of glutamine side chains in disease-related aggregation. Chem. Sci..

[236] Adegbuyiro A., Sedighi F., Pilkington A.W., Groover S., Legleiter J. (2017). Proteins Containing Expanded Polyglutamine Tracts and Neurodegenerative Disease. Biochemistry.

[237] Wen J., Scoles D.R., Facelli J.C. (2017). Molecular dynamics analysis of the aggregation propensity of polyglutamine segments. PLoS ONE.

[238] Babin V., Roland C., Sagui C. (2011). The α-sheet: A missing-in-action secondary structure?. Proteins.

[239] Adzhubei A.A., Sternberg MJ E., Makarov A.A. (2013). Polyproline-II Helix in Proteins: Structure and Function. J. Mol. Biol..

[240] Moradi M., Babin V., Roland C., Sagui C. (2012). Are Long-Range Structural Correlations Behind the Aggregration Phenomena of Polyglutamine Diseases?. PLoS Comput. Biol..

[241] Ruff K.M., Khan S.J., Pappu R.V. (2014). A Coarse-Grained Model for Polyglutamine Aggregation Modulated by Amphipathic Flanking Sequences. Biophys. J..

[242] Baskar M., Shafique M., Nandel F.S. (2014). A Unique Conformational Behaviour of Glutamine Peptides. J. Biophys. Chem..

[243] Chiang H., Chen C., Okumura H., Hu C. (2014). Transformation between α-helix and β-sheet structures of one and two polyglutamine peptides in explicit water molecules by replica-exchange molecular dynamics simulations. J. Comput. Chem..

[244] Chen S., Berthelier V., Yang W., Wetzel R. (2001). Polyglutamine aggregation behavior in vitro supports a recruitment mechanism of cytotoxicity. J. Mol. Biol..

[245] Robertson A.L., Horne J., Ellisdon A.M., Thomas B., Scanlon M.J., Bottomley S.P. (2008). The Structural Impact of a Polyglutamine Tract Is Location-Dependent. Biophys. J..

[246] Masino L. (2004). Polyglutamine and neurodegeneration: Structural aspects. Protein Pept. Lett..

[247] Papaleo E., Invernizzi G. (2011). Conformational diseases: Structural studies of aggregation of polyglutamine proteins. Curr. Comput. Aided Drug Des..

[248] Fiumara F., Fioriti L., Kandel E.R., Hendrickson W.A. (2010). Essential Role of Coiled Coils for Aggregation and Activity of Q/N-Rich Prions and PolyQ Proteins. Cell.

[249] Petrakis S., Schaefer M.H., Wanker E.E., Andrade-Navarro M.A. (2013). Aggregation of polyQ-extended proteins is promoted by interaction with their natural coiled-coil partners. Bioessays.

[250] Bhattacharyya A., Thakur A.K., Chellgren V.M., Thiagarajan G., Williams A.D., Chellgren B.W., Creamer T.P., Wetzel R. (2006). Oligoproline Effects on Polyglutamine Conformation and Aggregation. J. Mol. Biol..

[251] Pelassa I., Corà D., Cesano F., Monje F.J., Montarolo P.G., Fiumara F. (2014). Association of polyalanine and polyglutamine coiled coils mediates expansion disease-related protein aggregation and dysfunction. Hum. Mol. Genet..

[252] Schaefer M.H., Wanker E.E., Andrade-Navarro M.A. (2012). Evolution and function of CAG/polyglutamine repeats in protein–protein interaction networks. Nucleic Acids Res..

[253] Haaga J., Gunton J.D., Buckles C.N., Rickman J.M. (2018). Early stage aggregation of a coarse-grained model of polyglutamine. J. Chem. Phys..

[254] Mishra R., Thakur A.K. (2020). Exceptional Aggregation Propensity of Amino Acids in Polyglutamine Amino-Acid-Homopolymer. bioRxiv.

[255] Chiti F., Stefani M., Taddei N., Ramponi G., Dobson C.M. (2003). Rationalization of the effects of mutations on peptide and protein aggregation rates. Nature.

[256] Richardson J.S., Richardson D.C. (2002). Natural β-sheet proteins use negative design to avoid edge-to-edge aggregation. Proc. Natl. Acad. Sci. USA.

[257] Moldovean S.N., Chiş V. (2020). Specific Key-Point Mutations along the Helical Conformation of Huntingtin-Exon 1 Protein Might Have an Antagonistic Effect on the Toxic Helical Content’s Formation. ACS Chem. Neurosci..

[258] Moldovean S.N., Chiş V. (2021). Decreased Interactions between Calmodulin and a Mutant Huntingtin Model Might Reduce the Cytotoxic Level of Intracellular Ca^2+^: A Molecular Dynamics Study. Int. J. Mol. Sci..

[259] Feng M., Bell D.R., Wang Z., Zhang W. (2020). Length-Dependent Structural Transformations of Huntingtin PolyQ Domain Upon Binding to 2D-Nanomaterials. Front. Chem..

[260] Barrera E.E., Zonta F., Pantano S. (2021). Dissecting the role of glutamine in seeding peptide aggregation. Comput. Struct. Biotechnol. J..

[261] Zhou Z.-L., Zhao J.-H., Liu H.-L., Wu J.W., Liu K.-T., Chuang C.-K., Tsai W.-B., Ho Y. (2011). The Possible Structural Models for Polyglutamine Aggregation: A Molecular Dynamics Simulations Study. J. Biomol. Struct. Dyn..

[262] Pinheiro F., Santos J., Ventura S. (2021). AlphaFold and the amyloid landscape. J. Mol. Biol..

[263] Ourmazd A., Moffat K., Lattman E.E. (2022). Structural biology is solved—Now what?. Nat. Methods.

[264] Schleif R., Espinosa M. (2022). Where to From Here?. Front. Mol. Biosci..

[265] Lane T.J. (2023). Protein structure prediction has reached the single-structure frontier. Nat. Methods.

[266] Escobedo A., Piccirillo J., Aranda J., Diercks T., Topal B., Biesaga M., Staby L., Kragelund L.L., García J., Millet O. (2022). A Glutamine-Based Single ɑ-Helix Scaffold to Target Globular Proteins. bioRxiv.

[267] Warner J.B., Ruff K.M., Tan P.S., Lemke E.A., Pappu R.V., Lashuel H.A. (2017). Monomeric Huntingtin Exon 1 Has Similar Overall Structural Features for Wild-Type and Pathological Polyglutamine Lengths. J. Am. Chem. Soc..

[268] Escobedo A., Topal B., Kunze M.B.A., Aranda J., Chiesa G., Mungianu D., Bernardo-Seisdedos G., Eftekharzadeh B., Gairí M., Pierattelli R. (2019). Side chain to main chain hydrogen bonds stabilize a polyglutamine helix in a transcription factor. Nat. Commun..

[269] Krobitsch S., Lindquist S. (2000). Aggregation of huntingtin in yeast varies with the length of the polyglutamine expansion and the expression of chaperone proteins. Proc. Natl. Acad. Sci. USA.

[270] Chen S., Ferrone F.A., Wetzel R. (2002). Huntington’s disease age-of-onset linked to polyglutamine aggregation nucleation. Proc. Natl. Acad. Sci. USA.

[271] Gusella J.F., MacDonald M.E. (2006). Huntington’s disease: Seeing the pathogenic process through a genetic lens. Trends Biochem. Sci..

[272] Bates G.P., Dorsey R., Gusella J.F., Hayden M.R., Kay C., Leavitt B.R., Nance M., Ross C.A., Scahill R.I., Wetzel R. (2015). Huntington disease. Nat. Rev. Dis. Primers.

[273] Aziz N.A., Jurgens C.K., Landwehrmeyer G.B., van Roon-Mom W., van Ommen G.J., Stijnen T., Roos R.A., EHDN Registry Study Group (2009). Normal and mutant *HTT* interact to affect clinical severity and progression in Huntington disease. Neurology.

[274] Aziz N.A., van der Burg J.M., Tabrizi S.J., Landwehrmeyer G.B. (2018). Overlap between age-at-onset and disease-progression determinants in Huntington disease. Neurology.

[275] Jakubek R.S., Workman R.J., White S.E., Asher S.A. (2019). Polyglutamine Solution-State Structural Propensity Is Repeat Length Dependent. J. Phys. Chem. B.

[276] Leavitt B.R., Guttman J.A., Hodgson J., Kimel G.H., Singaraja R., Vogl A., Hayden M.R. (2001). Wild-Type Huntingtin Reduces the Cellular Toxicity of Mutant Huntingtin In Vivo. Am. J. Hum. Genet..

[277] Saleh A.A., Bhadra A.K., Roy I. (2014). Cytotoxicity of Mutant Huntingtin Fragment in Yeast Can Be Modulated by the Expression Level of Wild Type Huntingtin Fragment. ACS Chem. Neurosci..

[278] Sethi R., Patel V., Saleh A.A., Roy I. (2016). Cellular toxicity of yeast prion protein Rnq1 can be modulated by N-terminal wild type huntingtin. Arch. Biochem. Biophys..

[279] Sethi R., Tripathi N., Pallapati A.R., Gaikar A., Bharatam P.V., Roy I. (2018). Does N-terminal huntingtin function as a ‘holdase’ for inhibiting cellular protein aggregation?. FEBS J..

[280] Sethi R., Roy I. (2020). Stabilization of elongated polyglutamine tracts by a helical peptide derived from *N*-terminal huntingtin. IUBMB Life.

[281] Rockabrand E., Slepko N., Pantalone A., Nukala V.N., Kazantsev A., Marsh J.L., Sullivan P.G., Steffan J.S., Sensi S.L., Thompson L.M. (2007). The first 17 amino acids of Huntingtin modulate its sub-cellular localization, aggregation and effects on calcium homeostasis. Hum. Mol. Genet..

[282] Marquette A., Aisenbrey C., Bechinger B. (2021). Membrane Interactions Accelerate the Self-Aggregation of Huntingtin Exon 1 Fragments in a Polyglutamine Length-Dependent Manner. Int. J. Mol. Sci..

[283] Pandey N.K., Isas J.M., Rawat A., Lee R.V., Langen J., Pandey P., Langen R. (2018). The 17-residue-long N terminus in huntingtin controls stepwise aggregation in solution and on membranes via different mechanisms. J. Biol. Chem..

[284] Tam S., Spiess C., Auyeung W., Joachimiak L., Chen B., A Poirier M., Frydman J. (2009). The chaperonin TRiC blocks a huntingtin sequence element that promotes the conformational switch to aggregation. Nat. Struct. Mol. Biol..

[285] Thakur A.K., Jayaraman M., Mishra R., Thakur M., Chellgren V.M., Byeon I.-J.L., Anjum D.H., Kodali R., Creamer T.P., Conway J.F. (2009). Polyglutamine disruption of the huntingtin exon 1 N terminus triggers a complex aggregation mechanism. Nat. Struct. Mol. Biol..

[286] Kelley N.W., Huang X., Tam S., Spiess C., Frydman J., Pande V.S. (2009). The Predicted Structure of the Headpiece of the Huntingtin Protein and Its Implications on Huntingtin Aggregation. J. Mol. Biol..

[287] Boatz J.C., Piretra T., Lasorsa A., Matlahov I., Conway J.F., van der Wel P.C. (2020). Protofilament Structure and Supramolecular Polymorphism of Aggregated Mutant Huntingtin Exon 1. J. Mol. Biol..

[288] Michalek M., Salnikov E.S., Bechinger B. (2013). Structure and Topology of the Huntingtin 1–17 Membrane Anchor by a Combined Solution and Solid-State NMR Approach. Biophys. J..

[289] Michalek M., Salnikov E.S., Werten S., Bechinger B. (2013). Membrane Interactions of the Amphipathic Amino Terminus of Huntingtin. Biochemistry.

[290] Côté S., Binette V., Salnikov E.S., Bechinger B., Mousseau N. (2015). Probing the Huntingtin 1-17 Membrane Anchor on a Phospholipid Bilayer by Using All-Atom Simulations. Biophys. J..

[291] Kegel-Gleason K.B. (2013). Huntingtin Interactions with Membrane Phospholipids: Strategic Targets for Therapeutic Intervention?. J. Huntington’s Dis..

[292] Nagarajan A., Jawahery S., Matysiak S. (2014). The Effects of Flanking Sequences in the Interaction of Polyglutamine Peptides with a Membrane Bilayer. J. Phys. Chem. B.

[293] Tanaka Y., Igarashi S., Nakamura M., Gafni J., Torcassi C., Schilling G., Crippen D., Wood J.D., Sawa A., Jenkins N.A. (2006). Progressive phenotype and nuclear accumulation of an amino-terminal cleavage fragment in a transgenic mouse model with inducible expression of full-length mutant huntingtin. Neurobiol. Dis..

[294] Hackam A.S., Hodgson J.G., Singaraja R., Zhang T., Gan L., Gutekunst C.-A., Hersch S.M., Hayden M.R. (1999). Evidence for both nucleus and cytoplasm as subcellular sites of pathogenesis in Huntington’sdisease in cell culture and in transgenic mice expressing mutant huntingtin. Phil. Trans. R. Soc. Lond. B.

[295] Xia J. (2003). Huntingtin contains a highly conserved nuclear export signal. Hum. Mol. Genet..

[296] Choo Y.S. (2004). Mutant huntingtin directly increases susceptibility of mitochondria to the calcium-induced permeability transition and cytochrome c release. Hum. Mol. Genet..

[297] Liu Q., Huang S., Yin P., Yang S., Zhang J., Jing L., Cheng S., Tang B., Li X.-J., Pan Y. (2020). Cerebellum-enriched protein INPP5A contributes to selective neuropathology in mouse model of spinocerebellar ataxias type 17. Nat. Commun..

[298] Marcelo A., Afonso I.T., Afonso-Reis R., Brito D.V.C., Costa R.G., Rosa A., Alves-Cruzeiro J., Ferreira B., Henriques C., Nobre R.J. (2021). Autophagy in Spinocerebellar ataxia type 2, a dysregulated pathway, and a target for therapy. Cell Death Dis..

[299] Jain M., Patil N., Abdi G., Tarighat M.A., Mohammed A., Zain M.R.A.M., Goh K.W. (2023). Mechanistic Insights and Potential Therapeutic Approaches in PolyQ Diseases via Autophagy. Biomedicines.

[300] Zhao Y., Zurawel A.A., Jenkins N.P., Duennwald M.L., Cheng C., Kettenbach A.N., Supattapone S. (2018). Comparative Analysis of Mutant Huntingtin Binding Partners in Yeast Species. Sci. Rep..

[301] Dabrowska M., Juzwa W., Krzyzosiak W.J., Olejniczak M. (2018). Precise Excision of the CAG Tract from the Huntingtin Gene by Cas9 Nickases. Front. Neurosci..

[302] Shin J.W., Kim K.-H., Chao M.J., Atwal R.S., Gillis T., MacDonald M.E., Gusella J.F., Lee J.-M. (2016). Permanent inactivation of Huntington’s disease mutation by personalized allele-specific CRISPR/Cas9. Hum. Mol. Genet..

[303] Kolli N., Lu M., Maiti P., Rossignol J., Dunbar G. (2017). CRISPR-Cas9 Mediated Gene-Silencing of the Mutant Huntingtin Gene in an In Vitro Model of Huntington’s Disease. Int. J. Mol. Sci..

[304] Monteys A.M., Ebanks S.A., Keiser M.S., Davidson B.L. (2017). CRISPR/Cas9 Editing of the Mutant Huntingtin Allele In Vitro and In Vivo. Mol. Ther..

[305] Fiszer A., Krzyzosiak W.J. (2014). Oligonucleotide-based strategies to combat polyglutamine diseases. Nucleic Acids Res..

[306] Keiser M.S., Kordasiewicz H.B., McBride J.L. (2016). Gene suppression strategies for dominantly inherited neurodegenerative diseases: Lessons from Huntington’s disease and spinocerebellar ataxia. Hum. Mol. Genet..

[307] Gonzalez-Alegre P. (2019). Recent advances in molecular therapies for neurological disease: Triplet repeat disorders. Hum. Mol. Genet..

[308] Silva A.C., Lobo D.D., Martins I.M., Lopes S.M., Henriques C., Duarte S.P., Dodart J.-C., Nobre R.J., de Almeida L.P. (2020). Antisense oligonucleotide therapeutics in neurodegenerative diseases: The case of polyglutamine disorders. Brain.

[309] Tabrizi S.J., Leavitt B.R., Landwehrmeyer G.B., Wild E.J., Saft C., Barker R.A., Blair N.F., Craufurd D., Priller J., Rickards H. (2019). Targeting Huntingtin Expression in Patients with Huntington’s Disease. N. Engl. J. Med..

[310] Kwon D. (2021). Failure of genetic therapies for Huntington’s devastates community. Nature.

[311] Kingwell K. (2021). Double setback for ASO trials in Huntington disease. Nat. Rev. Drug Discov..

[312] Zeitler B., Froelich S., Marlen K., Shivak D.A., Yu Q., Li D., Pearl J.R., Miller J.C., Zhang L., Paschon D.E. (2019). Allele-selective transcriptional repression of mutant HTT for the treatment of Huntington’s disease. Nat. Med..

[313] Alterman J.F., Godinho B.M.D.C., Hassler M.R., Ferguson C.M., Echeverria D., Sapp E., Haraszti R.A., Coles A.H., Conroy F., Miller R. (2019). A divalent siRNA chemical scaffold for potent and sustained modulation of gene expression throughout the central nervous system. Nat. Biotechnol..

[314] Schuster K.H., Zalon A.J., Zhang H., DiFranco D.M., Stec N.R., Haque Z., Blumenstein K.G., Pierce A.M., Guan Y., Paulson H.L. (2022). Impaired Oligodendrocyte Maturation Is an Early Feature in SCA3 Disease Pathogenesis. J. Neurosci..

[315] Ingram M., Wozniak E.A., Duvick L., Yang R., Bergmann P., Carson R., O’callaghan B., Zoghbi H.Y., Henzler C., Orr H.T. (2016). Cerebellar Transcriptome Profiles of ATXN1 Transgenic Mice Reveal SCA1 Disease Progression and Protection Pathways. Neuron.

[316] Duvick L., Barnes J., Ebner B., Agrawal S., Andresen M., Lim J., Giesler G.J., Zoghbi H.Y., Orr H.T. (2010). SCA1-like Disease in Mice Expressing Wild-Type Ataxin-1 with a Serine to Aspartic Acid Replacement at Residue 776. Neuron.

[317] Hamel K., Moncada E.L., Sheeler C., Rosa J., Gilliat S., Zhang Y., Cvetanovic M. (2022). Loss of Intracerebellar Heterogeneity and Selective Vulnerability in Spinocerebellar Ataxia Type 1 Neurodegeneration. bioRxiv.

[318] Martinez-Rojas V.A., Juarez-Hernandez L.J., Musio C. (2022). Ion channels and neuronal excitability in polyglutamine neurodegenerative diseases. Biomol. Concepts.

[319] Coffin S.L., Durham M.A., Nitschke L., Xhako E., Brown A.M., Revelli J.-P., Gonzalez E.V., Lin T., Handler H.P., Dai Y. (2023). Disruption of the ATXN1-CIC complex reveals the role of additional nuclear ATXN1 interactors in spinocerebellar ataxia type 1. Neuron.

[320] Piol D., Tosatto L., Zuccaro E., Anderson E.N., Falconieri A., Polanco M.J., Marchioretti C., Lia F., White J., Bregolin E. (2023). Antagonistic effect of cyclin-dependent kinases and a calcium-dependent phosphatase on polyglutamine-expanded androgen receptor toxic gain of function. Sci. Adv..

[321] Pigazzini M.L., Lawrenz M., Margineanu A., Kaminski Schierle G.S., Kirstein J. (2021). An Expanded Polyproline Domain Maintains Mutant Huntingtin Soluble in vivo and During Aging. Front. Mol. Neurosci..

[322] Zhang L., Kang H., Perez-Aguilar J.M., Zhou R. (2022). Possible Co-Evolution of Polyglutamine and Polyproline in Huntingtin Protein: Proline-Rich Domain as Transient Folding Chaperone. J. Phys. Chem. Lett..

[323] Urbanek A., Popovic M., Elena-Real C.A., Morató A., Estaña A., Fournet A., Allemand F., Gil A.M., Cativiela C., Cortés J. (2020). Evidence of the Reduced Abundance of Proline cis Conformation in Protein Poly Proline Tracts. J. Am. Chem. Soc..

[324] Kapadia K., Trojniak A.E., Rodríguez K.B.G., Klus N.J., Huntley C., McDonald P., Roy A., Frankowski K.J., Aubé J., Muma N.A. (2022). Small-Molecule Disruptors of Mutant Huntingtin–Calmodulin Protein–Protein Interaction Attenuate Deleterious Effects of Mutant Huntingtin. ACS Chem. Neurosci..

[325] Truant R., Atwal R.S., Desmond C., Munsie L., Tran T. (2008). Huntington’s disease: Revisiting the aggregation hypothesis in polyglutamine neurodegenerative diseases. FEBS J..

[326] Nakamura K. (2001). SCA17, a novel autosomal dominant cerebellar ataxia caused by an expanded polyglutamine in TATA-binding protein. Hum. Mol. Genet..

[327] Punihaole D., Jakubek R.S., Workman R.J., Marbella L.E., Campbell P., Madura J.D., Asher S.A. (2017). Monomeric Polyglutamine Structures That Evolve into Fibrils. J. Phys. Chem. B.

[328] Wang X., Vitalis A., Wyczalkowski M.A., Pappu R.V. (2006). Characterizing the conformational ensemble of monomeric polyglutamine. Proteins.

[329] Hong J.-Y., Wang D.-D., Xue W., Yue H.-W., Yang H., Jiang L.-L., Wang W.-N., Hu H.-Y. (2019). Structural and dynamic studies reveal that the Ala-rich region of ataxin-7 initiates α-helix formation of the polyQ tract but suppresses its aggregation. Sci. Rep..

[330] Kandola T., Venkatesan S., Zhang J., Lerbakken B.T., Von Schulze A., Blanck J.F., Wu J., Unruh J.R., Berry P., Lange J.J. (2023). Pathologic Polyglutamine Aggregation Begins with a Self-Poisoning Polymer Crystal. Elife.

[331] Hatano Y., Ishihara T., Hirokawa S., Onodera O. (2023). Machine Learning Approach for the Prediction of Age-Specific Probability of SCA3 and DRPLA by Survival Curve Analysis. Neurol. Genet..

[332] Berman H., Henrick K., Nakamura H. (2003). Announcing the worldwide Protein Data Bank. Nat. Struct. Mol. Biol..

[333] Estevam B., Matos C.A., Nóbrega C. (2023). PolyQ Database—An integrated database on polyglutamine diseases. Database.

[334] Nóbrega C., Pereira De Almeida L. (2018). Polyglutamine Disorders.

